# A Reduction-Based Sensor for Acrolein Conjugates with the Inexpensive Nitrobenzene as an Alternative to Monoclonal Antibody

**DOI:** 10.1038/srep35872

**Published:** 2016-10-26

**Authors:** Masayuki Takamatsu, Koichi Fukase, Ritsuko Oka, Shinobu Kitazume, Naoyuki Taniguchi, Katsunori Tanaka

**Affiliations:** 1 Biofunctional Synthetic Chemistry Laboratory, RIKEN, 2-1 Hirosawa, Wako, Saitama 351-0198, Japan; 2 Department of Chemistry Graduate School of Science, Osaka University, 1-1 Machikaneyama, Toyonaka, Osaka 560-0043, Japan; 3 Disease Glycomics Team, Global Research Cluster, RIKEN-Max Planck Joint Research Center for Systems Chemical Biology, RIKEN, 2-1 Hirosawa, Wako, Saitama 351-0198, Japan; 4 Biofunctional Chemistry Laboratory, A. Butlerov Institute of Chemistry, Kazan Federal University, 18 Kremlyovskaya street, Kazan 420008, Russia; 5 Japan Science and Technology Agency-PRESTO, 2-1 Hirosawa, Wako, Saitama 351-0198, Japan

## Abstract

Acrolein, a highly toxic α, β–unsaturated aldehyde, has been a longstanding key biomarker associated with a range of disorders related to oxidative stresses. One of the most promising methods for detecting acrolein involves the use of antibodies that can recognize the acrolein–lysine conjugate, 3-formyl-3, 4-dehydropiperidines (FDP), within oxidatively stressed cells and tissues from various disease states. We have uncovered here that FDP could reduce nitroarenes in high yields at 100 °C in the presence of excess CaCl_2_ as a Lewis acid promoter. This unique transformation allowed for the development of a *de novo* method for detecting levels of FDPs generated from proteins in urine or blood serum samples. Thus we successfully converted a non-fluorescent and inexpensive 4-nitrophthalonitrile probe to the corresponding fluorescent aniline, thereby constituting the concept of fluorescent switching. Its sensitivity level (0.84 nmol/mL) is more than that of ELISA assays (3.13 nmol/mL) and is already equally reliable and reproducible at this early stage of development. More importantly, this method is cost effective and simple to operate, requiring only mixing of samples with a kit solution. Our method thus possesses potential as a future alternative to the more costly and operatively encumbered conventional antibody-based methods.

Acrolein is a highly toxic unsaturated aldehyde generated from an array of sources ranging from tobacco smoke to incomplete combustion of oil, charcoal, wood, plastic and other organic substances (Fig. 1A)^1^. It can also be produced endogenously under oxidative stresses from polyamines, lipids, amino acids and other biomolecules in the presence of reactive oxygen species^1^,^2^,^3^,^4^. Chemically, acrolein is well known to be highly reactive and have become recognized as a real hazard to the human health. More specifically, while reacting mainly with amino, thiol, and hydroxyl groups of proteins or nucleobases^5^,^6^, these intrinsic reactivities in biological settings often lead to many immunological disorders^7^,^8^ and inflammations^5^. Therefore, it remains critically important to understand relationships between acrolein and various diseases at the molecular level. Among these endeavors, developing new and improved detection of acrolein ranks at the very top in significance and urgency.

Detections of acrolein have traditionally been analyzed through chemical derivatizations with nucleophiles, i.e., 3-aminophenol^9^,^10^ or fluorescently labeled hydrazines^11^,^12^, although such methods require harsh conditions and often suffer from poor selectivity in the presence of other aldehydes. More sensitive and efficient methods that do not rely on HPLC, including a solid-supported two-reaction system, have been developed recently for detecting acrolein in mouse serum^13^. In our own work, we have managed to carry out the imaging of acrolein in oxidatively stressed cancer cells by means of unique acrolein/azide click reaction^14^. Acrolein exogenously or endogenously generated from oxidatively stressed cells could be directly visualized by simply treating cells with a fluorescent-azide probe. Alternatively, it has been known to diagnosis acrolein related diseases through detections of acrolein-amine conjugates^6^,^15^,^16^,^17^,^18^,^19^,^20^,^21^,^22^,^23^,^24^ such as 3-formyl-3,4-dehydropiperidine (or FDP; see **1** in Fig. 1A). Consequently, detection of FDP as biomarkers can indirectly indicate the biological concentration of acrolein^25^.

In addition to Igarashi’s elegant work^7^,^8^,^26^,^27^,^28^,^29^, the most notable method to date was reported by the Uchida’s laboratory involving immunochemical method (ELISA kit, Takara Bio Inc.)^6^,^15^,^25^,^30^,^31^. It is currently accepted to use monoclonal antibodies that recognize FDP-type structures in proteins. In particular, antibodies have been utilized for detecting of FDP-type structures to assess the disease state such as arterial sclerosis^15^,^32^,^33^,^34^, Alzheimer’s disease^35^,^36^, tumors^37^,^38^,^39^,^40^,^41^, diabetes^42^,^43^,^44^,^45^,^46^, autoimmune diseases^47^,^48^, hypertensions^27^, and many other types of diseases^49^,^50^,^51^,^52^. Uchida’s group also reported that oxidative stress markers include not only FDP (**1**) but also the pyridinium species such as **2**, which could be transformed from FDP via aromatic oxidation (Fig. 1A)^15^,^30^. This important finding implies that FDP also possess the reduction potential via hydrogen-transfer. Such assessment could find numerous precedents. For instance, nicotinamide adenine dinucleotide (NADH), a natural redox biomolecule that contains a similar dihydropyridine ring, can reduce ketones enzymatically (Fig. 1B)^53^. Similarly, vasicine is another natural product that has the reduction potential for nitro compounds through its own molecular oxidation (Fig. 1C)^54^. Given such reductive potential of FDP (**1**), we envisioned a possible fluorescent switch rendered by FDP during the conversion from non-fluorescent nitroarene probes to fluorescent anilines (Fig. 1D). This idea could make it possible to detect the acrolein-biomarkers easily without the use of the antibody. Herein, we wish to report a convenient and efficient method for detecting the oxidative stress marker FDP.

## Results and Discussion

**Figure 2:**
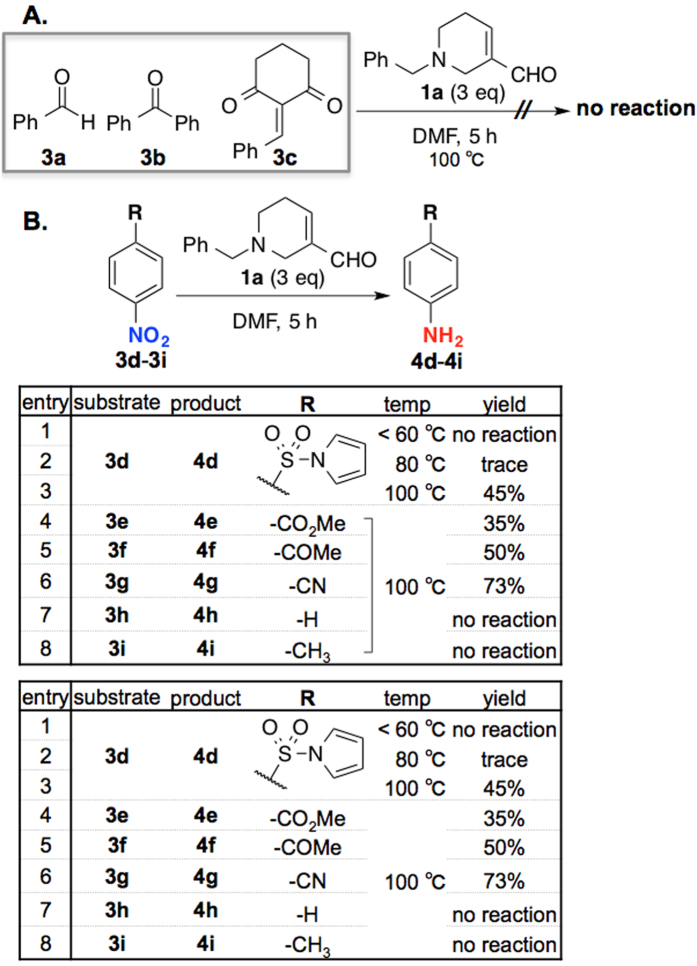
Initial attempts of reduction by *N*-Bn FDP 1a. (**A**) Reductions of simple aldehydes and ketones (**3a**–**c**). (**B**) Reductions of nitroarenes **3d**–**i**.

To commence our investigation, we attempted to use the readily accessible *N*-Bn-FDP (**1a**)^55^ as a reducing reagent against various substrates (Fig. 2). However, for examples containing carbonyl groups or unsaturated bond activated by an electron-withdrawing group (**3a**–**c**), reductions did not take place (Fig. 2A). On the other hand, we discovered that the nitroarene **3d** containing the sulfonyl group could be selectively reduced to the corresponding aniline **4d** using **1a** (Fig. 2B). While the reduction did not proceed under 60 °C (entry 1) initially with only trace amount of aniline **4d** was observed in the trial at 80 °C (entry 2), we found that 100 °C was the most optimal temperature for this reduction (entry 3, 45%). To examine the scope of this reduction, we proceeded with several commercially available nitroarenes as substrates. We found that like **3d** activated with an electron withdrawing sulfonyl group, nitrobenzene **3e** with an ester group could also reduced in 35% yield (entry 4), and that 4-nitroacetophenone **3f** and 4-nitrobenzonitrile **3g** were reduced in 50% and 73% yield, respectively (entries 5–6). On the other hand, nitrobenzenes **3h** and **3i** without an electron-withdrawing group were not reduced at all under the optimized conditions (entries 7–8).

**Figure 3:**
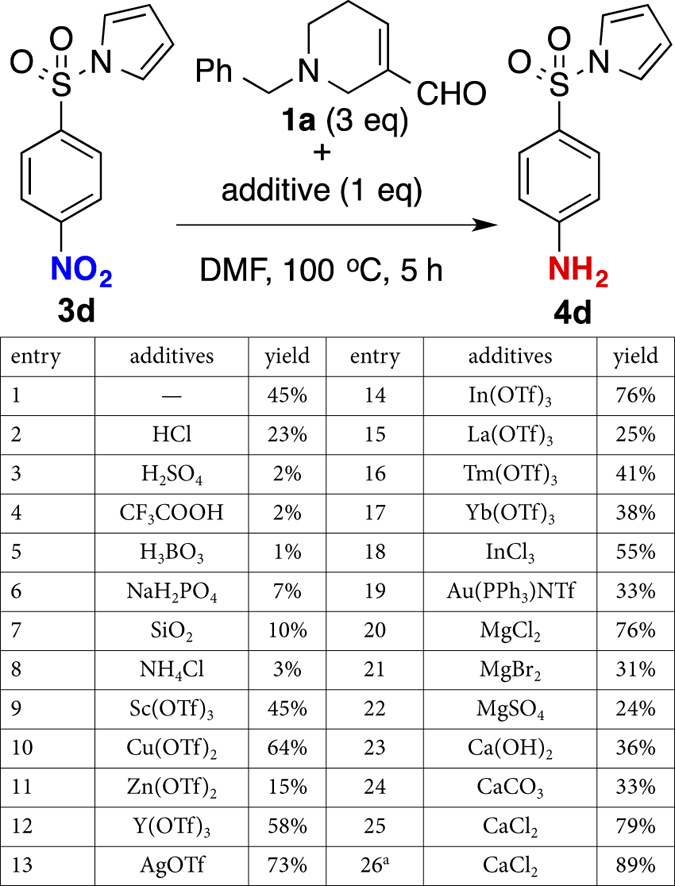
Evaluating additives for reduction. ^a^5 equiv of CaCl_2_ was added.

To improve the yield, we explored possibility of Brønsted acid or Lewis acid assisted reduction. Given that the NAD(P)H reduction can be promoted using a metal ion as catalyst^53^, we screened for a suitable acid using nitroarene **3d** as a model substrate (Fig. 3). Unfortunately, Brønsted acids such as HCl, H_2_SO_4_, CF_3_COOH, and H_3_BO_3_ (entry 2–8) led to decreased yields relative to our initial result (entry 1, 45%). Fortuitously, one equiv of transition metal-based Lewis acidic (e.g. Cu^2+^, In^3+^) additives led to a remarkable increase of the yields (entries 9–19, up to 76%). We particularly pleased to find that focused on alkaline earth metal-based Lewis acid (Mg^2+^, Ca^2+^), inspired by NADH reduction catalyzed by metal ion^53^, were equally effective (entries 20–25). With 5 equiv of CaCl_2_ being the most effective for this reduction at 89% yield (entry 26). Counter anion dependency on reactivity (among MgCl_2_, MgBr_2_, MgSO_4_, Ca(OH)_2_, CaCO_3_ and CaCl_2_) is most probably due to the solubility of metal species in reaction media; While MgCl_2_ and CaCl_2_ are soluble in either DMF or DMF/H_2_O, other species are hardly soluble or insoluble (see Fig. S1 in Supplementary Information). We also checked pH of the reaction in the presence of these metal species (Fig. S1). Although additives slightly affected solution pH, the observed reactivity in Fig. 3 could be well explained by the solubility of additives. For detecting FDPs in biological samples, i.e., urine (*vide infra*), very little amount of samples were diluted with excess H_2_O and then subjected to excess nitrobenzene and metal additives. Hence pH of sample solution should not be significantly concerned for our FDP detection.

**Figure 4:**
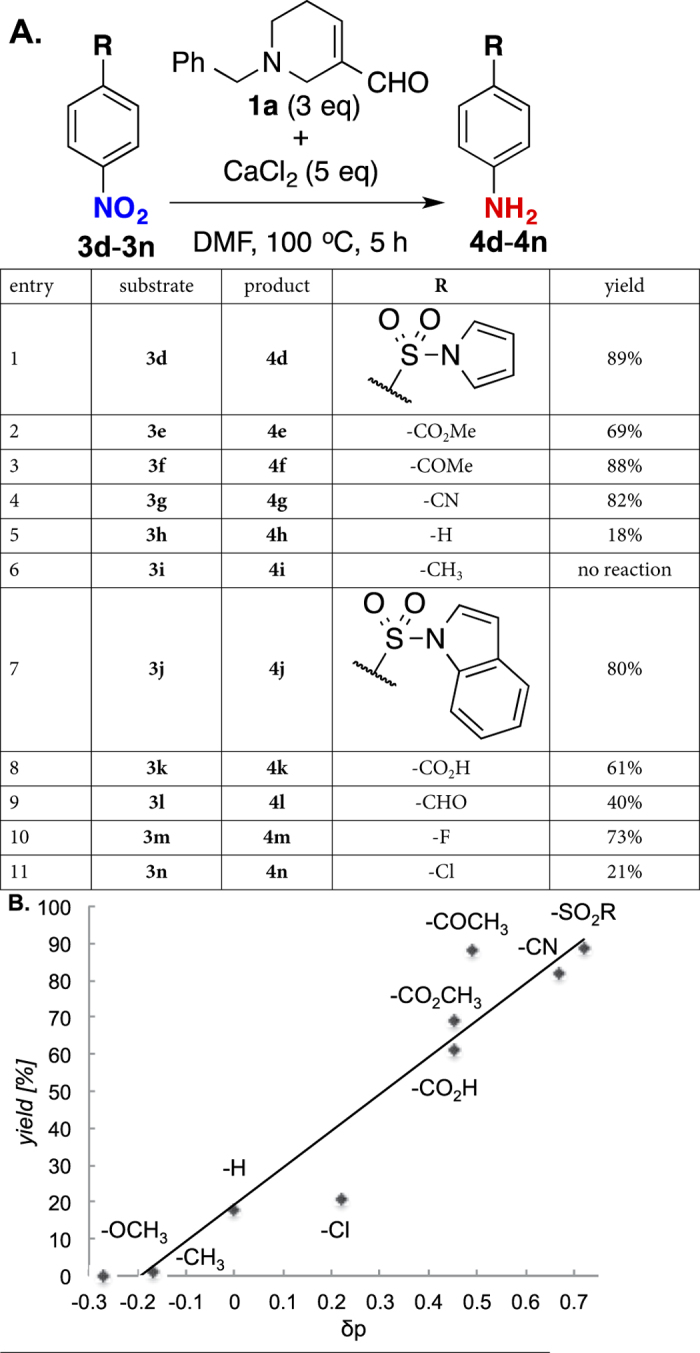
Substitution effects of reduction of nitrobenzene. (**A**) Conversion from nitroarenes **3d**–**n** to corresponding anilines **4d**–**n**. (**B**) Hammett plot of reduction yields against substituent groups.

Having established the optimized conditions (5 equiv of CaCl_2_, 100 °C, and 5 h), we proceeded to examine scope and limitation of this reduction with various nitroarenes. As shown in Fig. 4A, in comparison with the initial trial shown in Fig. 2B, nitrobenzenes containing sulfonyl (**3d**), ester (**3e**), ketone (**3f**), and nitrile (**3g**) as electron withdrawing groups were all reduced to corresponding anilines in higher yields (Fig. 4A, entries 1–4, **4d**: 89%, **4e**: 69%, **4f**: 88%, **4g**: 82%), thereby accentuates the importance of CaCl_2_. More notably, while simple nitrobenzene **3h** was not reduced at all in previous trial (Fig. 2B, entry 7), it could now be reduced to the corresponding aniline **4h** in 18% yield in the presence of CaCl_2_ (entry 5). On the other hand, 4-nitrotoluene **3i** containing an electro-donating group was still not reduced under these optimized conditions (entry 6). Nitrobenzene **3j** containing an indolyl sulfone motif, which is an intermediate in the indole alkaloid synthesis, could be reduced to **4j** in 80% yield (entry 7). Predictably, 4-nitrobenzoic acid **3k** and 4-nitrobenzaldehyde **3l** were reduced in 61% and 40%, respectively (entries 8–9). Similarly, 4-fluoronitrobenzene **3m** were naively reduced without any substitution (entry 10, 73%). The reduction yield was significantly lower when using 4-chloro-nitrobenzene **3n** with just an inductive electron-withdrawing group, although unreacted **3n** could be mostly recovered without any other isolable byproducts (entry 11, 21%). The unique substituent effect observed here is further supported by the linear Hammett plot shown in Fig. 4B.

To develop this reduction into an effective and simple method for FDP detection, we investigated possible reactions of FDP in biological sample with a non-fluorescent nitroarene probe affording a reaction mixture that contains fluorescence of the converted corresponding aniline. Conceptually, we envisioned that the fluorescence intensity should be proportional to the amount of the aniline, which is related with the amount of FDP at a constant rate via the reduction, and thus, the exact amount of FDP in biological sample could be estimated. Most critically for this endeavor, an adequate nitroarene probe is to detect the level of FDP in the presence of various kinds of biological materials. We focused on two points for selecting an appropriate nitroarene probe: (a) fluorescence property for switching; and (b) efficient reactivity.

**Figure 5:**
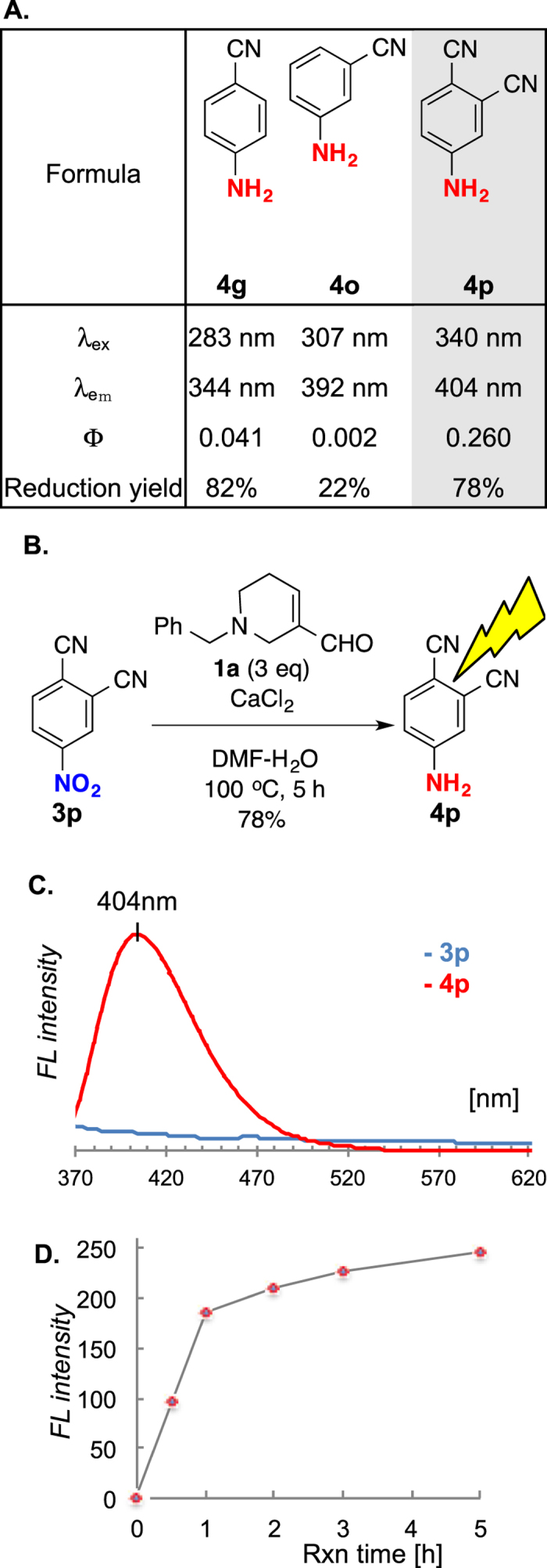
(**A**) Fluorescence and reactivity profiles of cyano-substituted anilines. (**B**) Reduction of 4-nitrophthalonitrile (**3p**). (**C**) Fluorescent spectrum of **3p** (blue) and **4p** (red) at 340 nm excitation. (**D**) Time-dependent analysis of fluorescent intensity by FDP reduction. Mean values with standard deviations are indicated. λ_ex_ = excitation wavelength, λ_em_ = emission wavelength, Φ = quantum yield.

With these assessments in mind, we proceeded to screen for nitroarenes that could be reduced to fluorescent anilines. Typical fluorescent anilines are classified electronically as being pull-push. That is, the aromatic ring is substituted with both electro-withdrawing and electron-donating groups such as the combination of nitrile and amino group, which is widely known to fluoresce (Fig. 5A)^56^. It is equally important that excitation and emission wavelengths are sufficiently resolved (non-overlapping), and that the quantum yield is sufficiently high for to an accurate measurement. After screening dozens of candidates including **4g** and **4o**, we found that 4-nitrophthalonitrile **3p** to be the most suitable nitroarene probe. As shown in Fig. 5B, while the reduction of **3p** is 78% yield and is complete in 5 h, the overlaid fluorescent spectrum of **3p** and **4p** suggested a clear switch at emission λmax of 404 nm (Fig. 5C). Moreover, time-dependent measurement reveals a distinct correlation between the fluorescence intensity and the reaction progress (Fig. 5D).

**Figure 6:**
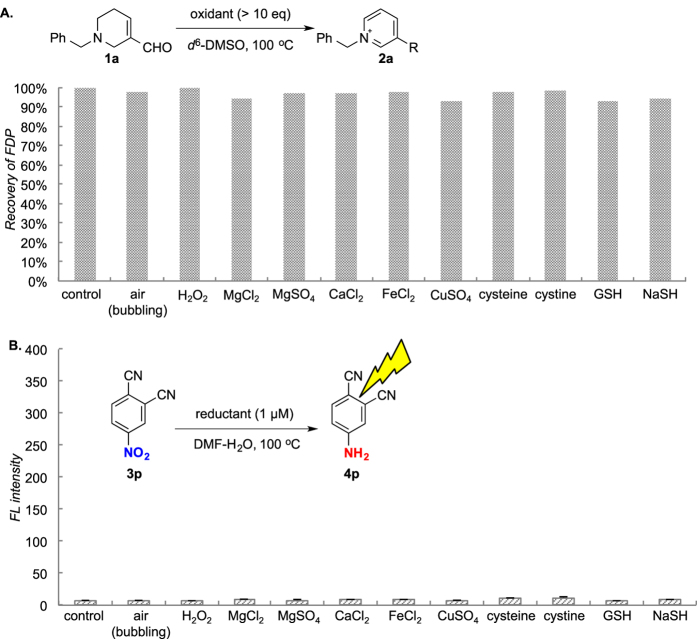
(**A**) Stability of FDP in the presence of various metals and redox reagents (air, H_2_O_2,_ MgCl_2_, MgSO_4_, CaCl_2_, FeCl_2_, CuSO_4_, cysteine, cystine, GSH, NaSH). Recovery ratio was determined by ^1^H NMR using an internal standard (dimethylsulfone). (**B**) Stability of nitrobenzene **3p** in the presence of various metals and redox reagents. Conversion was estimated by aniline fluorescence. Mean values with standard deviations are indicated.

To confirm the reduction-based “fluorescent on” concept for FDP, and to verify chemoselectivity of such concept under biological conditions, we tested robustness of FDP and nitroarene probe in the presence of biologically relevant metals and redox reagents (Fig. 6). Addition of air or hydrogen peroxide afforded no oxidation of FDP **1a** (Fig. 6A) and, in the presence of biologically abundant or redox metal species (Mg^2+^, Ca^2+^, Fe^2+^, Cu^2+^), FDP was recovered quantitatively. For the nitroarene probe, **3p** did not react with cystein, cystine, glutathione (GSH) or sodium hydrogen sulfide (NaSH) at 1 μM, which is supposed as biological concentration^57^ (Fig. 6B). Therefore, any biologically relevant metal species and redox agents gave little influence on FDPs and 4-nitrophthalonitrile **3p** themselves. Hence undesired consumption of FDPs or background fluorescence increase can be avoided under the established conditions. Namely, Ca^2+^ or Mg^2+^ selectively activate the reduction in the presence of both substrates. We do not think that very small amount of metal species existing in biological systems, in comparison to excess CaCl_2_ used in this research, can efficiently mediate the reaction, but even if so, this is advantageous for our reduction-based sensor.

**Figure 7:**
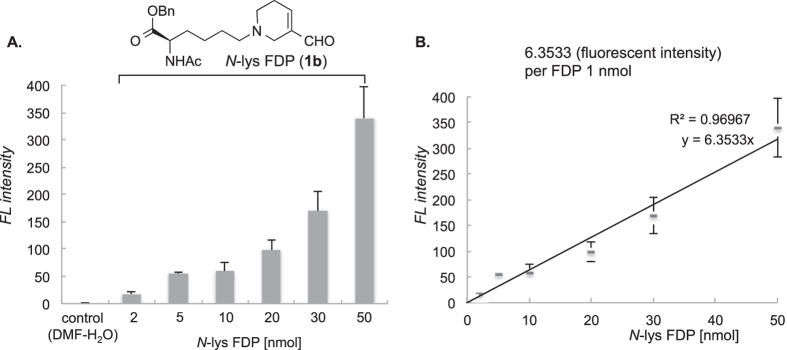
(**A**) Fluorescence intensity by prepared *N*-lys FDP **1b** solution (0, 2, 5, 10, 20, 30, 50 nmol/mL) as authentic standard in detection protocol. (**B**) Fluorescence intensity per FDP unit was calculated. Reaction was conducted under optimized kit condition (**3p**: 1.7 mg; CaCl_2_: 5.5 mg; temp: 100 °C; time: 5 h). Mean values with standard deviations are indicated.

To further demonstrate the reliability of this new detection method, we demonstrated biological application using *N*-lys FDP **1b** under established conditions as shown above. We established the precise correlation between the amount of FDP and the fluorescent intensity using authentic *N*-lys FDP as the standard (Fig. 7A). The delta values between each fluorescence and control are proportional to that of FDP amount (Fig. 7B). The slope of the linear plot in Fig. 7B indicates fluorescent intensity per FDP unit. According to statistical processing (see calculation in Supplementary Information, Fig. S2), detection limit (LOD) is determined to be 0.84 nmol/mL using this method, which is more sensitive than that of ELISA kit (3.13 nmol/mL).

**Figure 8:**
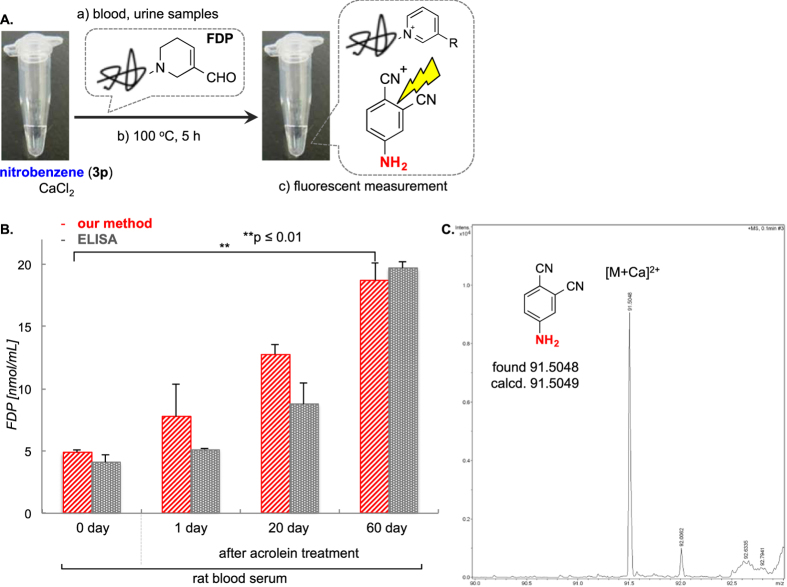
(**A**) Protocol of our detection method to mouse and rat blood samples. (**B**) Detection using rat blood serum treated with acrolein over 0 (normal), 1, 20, or 60 days and compared with the ELISA method. Mean values with standard deviations are indicated. **p < 0.01; p values were determined by using two-tailed Student *t* test. (**C**) Detection of **4p** by ESI-MS after reduction by 60 days’ sample. HRESI-MS *m/z* calcd for C_8_H_5_N_3_Ca [M+Ca]^2+^ 91.5049, found 91.5048.

With these validations in hand, we simplified the method by constructing a kit based on the optimized conditions. The procedure of detection is quite simple with three steps: (a) Mixing the sample and the pre-prepared kit solution containing 1.7 mg of nitroarene probe, and 5.5 mg of CaCl_2_ in 50 μL of DMF-H_2_O; (b) heating at 100 °C for 5 h; and c) measuring the resulting fluorescence (Fig. 8A).

We first evaluated a normal blood serum and prepared serum samples that contain artificially generated FDP via pre-treatment of excess acrolein over 0, 1, 20, and 60 days (Fig. 8B). We would measure the amount of FDP estimated by standard values as calculated in Fig. 7B. The rat blood serum normally has 4.9 ± 0.2 nmol/mL of FDP, which is what we find here. In comparison with this control, 1-day treatment of excess acrolein led to a little increase of FDP (7.7 ± 2.6 nmol/mL), but it the level was 12.7 ± 0.7 nmol/mL in the sample after 20-d treatment. In addition, a 60-d sample shows further increase of FDP level to 18.7 ± 1.4 nmol/mL. Fluorescent aniline **4p**, which was obtained by reduction with FDP in rat blood serum (pretreated with aclorein over 60 days, Fig. 8B), was successfully identified by ESI-MS (Fig. 8B). These results are in good agreement with ELISA assay of antibody recognizing FDP.

**Figure 9:**
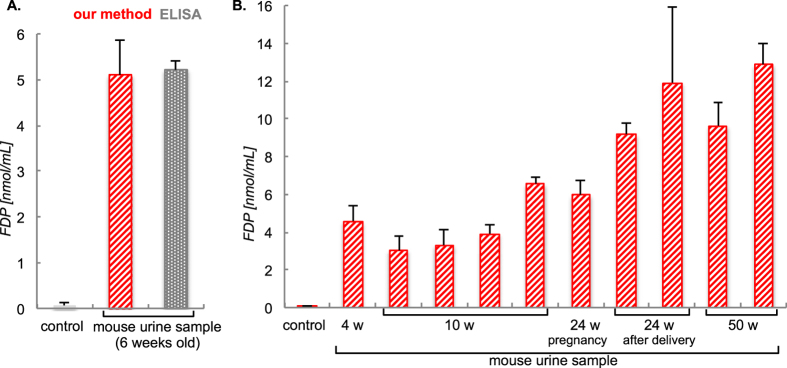
(**A**) Detection of FDP using the 20-fold diluted urine sample of a 6-week old mouse and compared with the ELISA method. (**B**) Simultaneous detection urine samples from ten mice (4, 10, 24, or 50 weeks old). Mean values with standard deviations are indicated.

We then pursued a more practical experiment by using a real urine sample from a 6-week old mouse. After preparation of 20-fold diluted urine sample and dividing the sample into three sample-lots, we attempted the FDP-detection using both our method in comparison with the ELISA protocol. As shown in Fig. 9A, the diluted urine sample showed a level of FDP of 5.2 ± 0.8 nmol/mL, which is in excellent statistical agreement with the ELISA assay^15^.

Finally, we conducted the detection urine samples from 10 mice (Fig. 9B). Although we conducted these experiment in triplicates (totally 30 lots), the entire set of urine test was carried out smoothly and swiftly using the kit solution within 6 h unlike any medical/laboratory services currently in practice. Our results show that levels of FDP in diluted urines from the 10-mice set are within the 3.4–13.1 nmol/mL range. We have thus succeeded in achieving a sensitive and inexpensive detection of FDP from biological samples extracted from mammalian models through the use of an under explored reaction of FDP.

## Conclusion

We have uncovered here *N*-Bn-3-formyl-3,4-dehydropiperidine, an analog of oxidative stress marker FDP, could reduce nitroarene in high yields using CaCl_2_ as a Lewis acid promoter. Based on this unique transformation, we developed a *de novo* detection method for FDP levels via converting a non-fluorescent nitroarene probe to the corresponding fluorescent aniline, thereby constituting the concept of fluorescent switching. This new method is amenable for actual biological samples; its sensitivity level is comparable to that of ELISA assays and is already equally reliable and reproducible at this early stage of development. More importantly, it is easy to handle, practical to operate, and cost effective. Biological samples containing specific nitrobenzenes, i.e., drugs with strongly electron-withdrawing groups, may react with FDP and affect the analysis. While method should be used with cautions, excess 4-nitrophthalonitrile **3p** with CaCl_2_ is otherwise preferentially reduced, hence could be applied in most cases for detecting oxidatively stressed diseases. Thus, our method possesses potential as a future alternative to the more costly and operatively encumbered conventional methods. Efforts are underway to further develop this novel detection method.

## Experimental Methods

### General information

All solvents were of reagent grade. All commercially purchased chemicals were used as received^1^. H and ^13^C NMR spectra were obtained from a JEOL RESONANCE AL400 NMR and a JEOL RESONANCE AL300 NMR spectrometer. Signals were internally referenced to solvent residues. High-resolution mass spectral analyses were carried out using micrOTOF-Q III-HC^TM^ (BRUKER). All fluorescent measurement was carried out using JASCO FP6500 spectrofluorometer with 96-well flat-bottomed plates from Corning Inc. Each value of amount is calculated from intensity of authentic standards. All procedures involving experiment animals was approved by the Ethics Committee of RIKEN. The experiments were performed in accordance with the institutional and national guidelines.

### A General Procedure for the Reduction of Nitroarenes with FDP

To a solution of nitoroarene **3d** (25.0 mg, 0.10 mmol) and CaCl_2_ (55.0 mg, 0.50 mmol) in DMF (0.2 mL) was added N-benzyl 3‒formyl‒3,4-dehydropiperidine (N-Bn FDP, 60.0 mg, 0.30 mmol) at 100 °C. After stirring for several hours at this temperature, the mixture was concentrated *in vacuo* to give a crude mixture as sticky gum. The crude residue was monitored in NMR or purified by either preparative TLC or silica gel flash column chromatography with the hexane-EtOAc solvent mixture as the eluting system to give the desired aniline product **4d** (19.6 mg, 89% yield).

### Sample Preparation and Detection Procedure

Normal rat serum (100 μL) purchased from Wako Pure Chemical Industry Ltd. was treated with acrolein 100 μL (>100-fold equivalent to serum proteins) for a specific number of days (0, 1, 20, or 60 d). After which, the sample was diluted to 1 mL with distilled water. Fresh urine samples (100 μL) were supplied from C57BL/6 mouse of RIKEN bio-resource center and diluted 20-fold with distilled water. To a solution of nitroarene probe **3k** (1.7 mg, 10.0 μmol) and CaCl_2_ (5.5 mg, 50.0 μmol) in DMF-H_2_O (50 μL) was added a given urine sample. After stirring for 5 h at 100 °C, the crude reaction mixture was filtered, and the resulting filtrate was measured by spectrofluorometer at 340 nm/404 nm.

### ELISA Assay

The measurement was conducted with the Enzyme Linked Immunosorbent Assay (ELISA) kit system (TAKARA, Acrolein-Lysine adduct competitive ELISA kit)^15^ and followed by attached instructions. The absorbance at 450 nm was measured using micro plate reader (ImmunoMini NJ1000). Data represent averages of more than twice assay with standard deviations from individual experiments.

### Characterizations

1-((4-aminophenyl)sulfonyl)-1*H*-pyrrole (**4d**, 89%): ^1^H NMR (400 MHz, CDCl_3_) δ 7.63 (dd, 2H, *J* = 6.7, 2.0 Hz), 7.13 (apparent t, 2H, *J* = 2.3 Hz), 6.63 (dd, 2H, *J* = 6.8, 2.0 Hz), 6.26 (apparent t, 2H, *J* = 2.3 Hz), 4.21 (brs, NH_2_); ^13^C NMR (100 MHz, CDCl_3_)δ 151.6, 129.2(2C), 126.8, 120.6(2C), 114.1(2C), 113.1(2C); HRESI-MS *m/z* calcd for C_10_H_10_N_2_O_2_S [M + H]^+^ 223.0536, found 223.0534.

Methyl 4-aminobenzoate (**4e**, 69%): ^1^H NMR (400 MHz, CDCl_3_) δ 7.85 (dd, 2H, *J* = 8.7, 2.8 Hz), 6.64 (dd, 2H, *J* = 8.7, 2.8 Hz), 4.10 (brs, NH_2_), 3.87 (s, 3H); ^13^C NMR (100 MHz, CDCl_3_) δ 167.2, 150.8, 131.2(2C), 119.7, 113.9(2C), 51.6; HRESI-MS *m/z* calcd for C_8_H_10_N_1_O_2_ [M + H]^+^ 152.0706, found 152.0709.

4-aminoacetophenone (**4f**, 88%): ^1^H NMR (400 MHz, CDCl_3_) δ 7.79 (dd, 2H, *J* = 8.6, 1.4 Hz), 6.63 (dd, 2H, *J* = 8.7, 1.4 Hz), 4.23 (brs, NH_2_), 2.49 (s, 3H); ^13^C NMR (100 MHz, CDCl_3_) δ 196,7, 151.3, 130.9(2C), 127.7, 119.7, 113.7(2C), 26.1; HRESI-MS *m/z* calcd for C_8_H_9_N_1_O_1_ [M + H]^+^ 136.0762, found 136.0757.

4-aminobenzonitrile (**4g**, 82%): ^1^H NMR (400 MHz, CDCl_3_) δ 7.41 (dd, 2H, *J* = 8.6, 1.2 Hz), 6.64 (dd, 2H, *J* = 8.6, 1.2 Hz), 4.17 (brs, NH_2_); ^13^C NMR (100 MHz, CDCl_3_) δ 150.5, 133.9(2C), 120.2, 114.6(2C), 100.4; HRESI-MS *m/z* calcd for C_7_H_6_N_2_ [M + H]^+^ 119.0604, found. 119.0605.

aniline (**4h**, 18%): ^1^H NMR (400 MHz, CDCl_3_) δ 7.13 (ddd, 2H, J = 7.5, 7.5, 1.2 Hz), 6.74 (tt, 1H, J = 7.5, 1.2 Hz), 6.65 (dd, 2H, J = 7.5, 1.2 Hz), 3.51(brs, NH_2_); ^13^C NMR (100 MHz, CDCl_3_) δ 146.3, 129.2(2C), 118.4, 115.0(2C).

1-((4-aminophenyl)sulfonyl)-1*H*-indole (**4j**, 80%): ^1^H NMR (400 MHz, CDCl_3_) δ 7.97 (dd, 1H, *J* = 8.3, 1.2 Hz), 7.66 (dd, 2H, *J* = 9.1, 2.4 Hz), 7.55(d, 1H, *J* = 3.6 Hz), 7.52 (d, 1H, *J* = 8.3 Hz), 7.29 (ddd, 1H, *J* = 8.3, 8.3, 1.2 Hz), 7.20 (ddd, 1H, *J* = 8.3, 8.3, 1.2 Hz), 6.62 (d, 1H, *J* = 3.6 Hz), 6.55 (dd, 2H, *J* = 9.1, 2.4 Hz), 4.11(brs, NH_2_); ^13^C NMR (100 MHz, CDCl_3_) δ 153.5, 151.3, 134.8, 130.7, 129.1 (2C), 126.3 (2C), 124.2, 122.9, 121.2, 113.9, 113.5, 108.4; HRESI-MS *m/z* calcd for C_14_H_13_N_2_O_2_S [M + H]^+^ 273.0692, found 273.0691.

4-aminobenzoic acid (**4k**, 61%): ^1^H NMR (400 MHz, *d*^*6*^-DMSO) δ 7.62 (d, 2H, *J* = 8.4 Hz), 6.54 (dd, 2H, *J* = 8.4 Hz), 5.89 (brs, NH_2_); ^13^C NMR (100 MHz, CDCl_3_) δ 167.6, 153.2, 131.3(2C), 116.9, 112.6(2C); HRESI-MS *m/z* calcd for C_7_H_7_N_1_O_2_ [M + H]^+^ 138.0550, found 138.0539.

4-aminobenzaldehyde (**4l**, 40%): ^1^H NMR (400 MHz, *d*^*6*^-DMSO) δ 9.56 (s, 1H), 7.54 (d, 2H, *J* = 8.7 Hz), 6.62 (d, 2H, *J* = 8.7 Hz), 4.14; ^13^C NMR (100 MHz, *d*^*6*^-DMSO) δ 189.7, 155.3(2C), 132.2, 124.8, 113.1(2C); HRESI-MS *m/z* calcd for C_7_H_7_N_1_O_1_ [M + H]^+^ 122.0600, found 122.0610.

4-fluoroaniline (**4m**, 73%): ^1^H NMR (400 MHz, CDCl_3_) δ 6.84–6.78 (m, 2H), 6.56–6.51 (m, 2H), 3.51(brs, NH_2_); ^13^C NMR (100 MHz, CDCl_3_) δ 156.1(d, J = 235 Hz, C-F), 142.4, 115.8(2C), 115.3(2C); HRESI-MS *m/z* calcd for C_6_H_6_F_1_N_1_ [M + H]^+^ 112.0557, found 112.0558.

4-chloroaniline (**4n**, 21%): ^1^H NMR (400 MHz, CDCl_3_) δ 7.09 (d, 2H, *J* = 8.6 Hz), 6.58 (d, 2H, *J* = 8.6 Hz), 3.45(brs, NH_2_); ^13^C NMR (100 MHz, CDCl_3_) δ 145.4, 129.2(2C), 123.2, 116.3(2C); HRESI-MS *m/z* calcd for C_6_H_6_N_1_Cl_1_ [M + H]^+^ 128.0262, found 128.0263.

3-aminobenzonitrile (**4o**, 22%): ^1^H NMR (300 MHz, CDCl_3_) δ 7.16 (dd, 1H, *J* = 7.2, 7.2 Hz), 6.94 (d, 1H, *J* = 7.2 Hz), 6.83–6.77 (m, 2H), 3.82 (brs, NH_2_); ^13^C NMR (75 MHz, CDCl_3_) δ 147.0, 130.2(2C), 122.1, 119.3, 117.6, 113.1; HRESI-MS *m/z* calcd for C_7_H_6_N_2_ [M + H]^+^ 119.0604, found. 119.0612.

4-aminophthalonitrile (**4p**, 78%): ^1^H NMR (400 MHz, *d*^*6*^-DMSO) δ 7.64 (d, 1H, *J* = 8.7 Hz), 7.03 (d, 1H, *J* = 2.4 Hz), 6.68 (dd, 1H, *J* = 8.7, 2.4 Hz), 6.72 (brs, NH_2_); ^13^C NMR (100 MHz, *d*^*6*^-DMSO) δ 153.2, 135.1, 117.6, 117.3, 117.1, 116.5, 115.6, 97.9; HRESI-MS *m/z* calcd for C_8_H_5_N_3_ [M + Na]^+^ 166.0400, found 166.0380. Fluorescent excitation/emission: 304/404 nm, quantum yield Φ = 0.26.

## Additional Information

**How to cite this article**: Takamatsu, M. *et al*. A Reduction-Based Sensor for Acrolein Conjugates with the Inexpensive Nitrobenzene as an Alternative to Monoclonal Antibody. *Sci. Rep.* **6**, 35872; doi: 10.1038/srep35872 (2016).

**Publisher’s note:** Springer Nature remains neutral with regard to jurisdictional claims in published maps and institutional affiliations.

## Supplementary Material

Supplementary Information

## Figures and Tables

**Figure 1:**
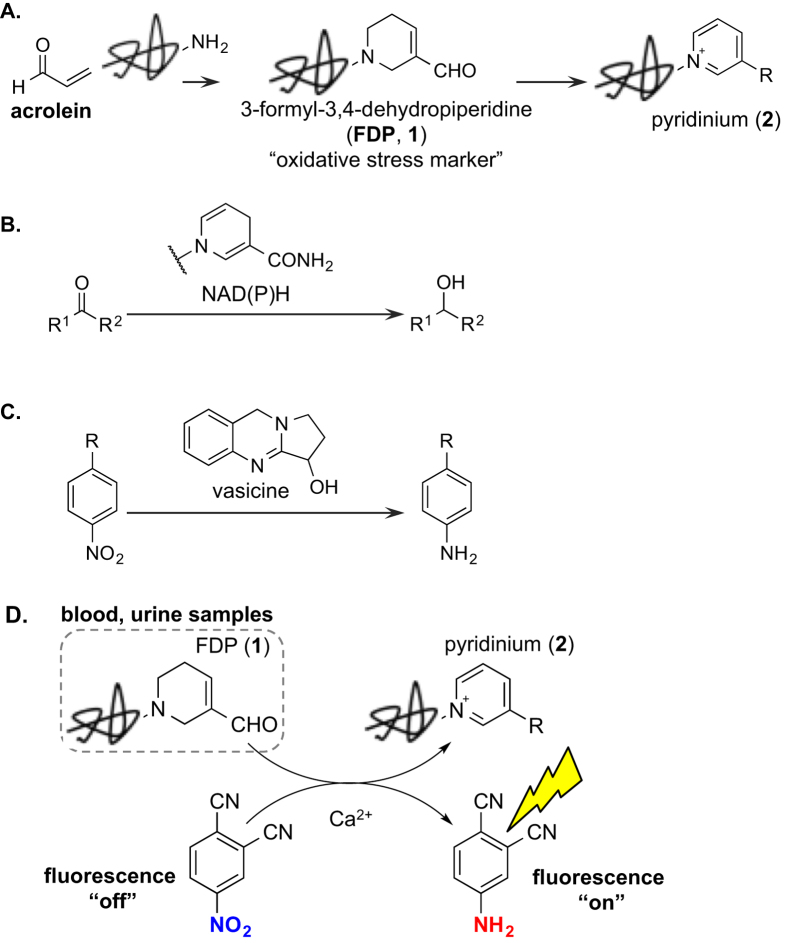
(**A**) Production of acrolein-conjugates (FDP, **1**) and their pyridinium derivatives (**2**). (**B**) Reduction by NAD(P)H. (**C**) Reduction by vacisine. (**D**) Detection of FDP using reduction of non-fluorescent nitroarenes to fluorescent anilines.
