# Supplementary material for: A Reduction-Based Sensor for Acrolein Conjugates with the Inexpensive Nitrobenzene as an Alternative to Monoclonal Antibody
Source: Sci Rep. 2016 Oct 26;6:35872. doi: 10.1038/srep35872 (PMC5080631; doi:10.1038/srep35872)
Supplement: Supplementary Information [file srep35872-s1.pdf]

Supplementary Information

A Reduction-Based Sensor for Acrolein Conjugates with the Inexpensive Nitrobenzene as an Alternative to Monoclonal Antibody.

Masayuki Takamatsu[1,2], Koichi Fukase[2], Ritsuko Oka[3], Shinobu Kitazume[3], Naoyuki Taniguchi[3] and Katsunori Tanaka[1,4,5,*]

[1]Biofunctional Synthetic Chemistry Laboratory, RIKEN, 2-1 Hirosawa, Wako, Saitama 351-0198, Japan

[2]Department of Chemistry Graduate School of Science, Osaka University, 1-1 Machikaneyama, Toyonaka, Osaka 560-0043, Japan

[3]Disease Glycomics Team, Global Research Cluster, RIKEN-Max Planck Joint Research Center for Systems Chemical Biology, RIKEN, 2-1 Hirosawa, Wako, Saitama 351-0198, Japan

[4]Biofunctional Chemistry Laboratory, A. Butlerov Institute of Chemistry, Kazan Federal University, 18 Kremlyovskaya street, Kazan 420008, Russia

[5]Japan Science and Technology Agency-PRESTO, 2-1 Hirosawa, Wako, Saitama 351-0198, Japan

*To whom correspondence should be addressed:

Biofunctional Synthetic Chemistry Laboratory, RIKEN, 2-1 Hirosawa, Wako, Saitama 351-0198, Japan.

Tel: (+81)-48-467-9405; Fax: (+81)-48-467-9379; E-mail: kotzenori@riken.jp (Katsunori Tanaka)

	DMF		$DMF-H_2O$	
additives	pH at 100 °C	solubility	solubility	reduction yield
$MgCl_2$	6.92	O	O	76%
$MgBr_2$	7.39	△	O	31%
$MgSO_4$	7.57	△	O	24%
$Ca(OH)_2$	11.61	X	△	36%
$CaCO_3$	8.63	X	X	33%
$CaCl_2$	7.24	O	O	79%

Figure S1. Correlation of reduction yields with solution pH and solubility of additives under reaction conditions in Table2.

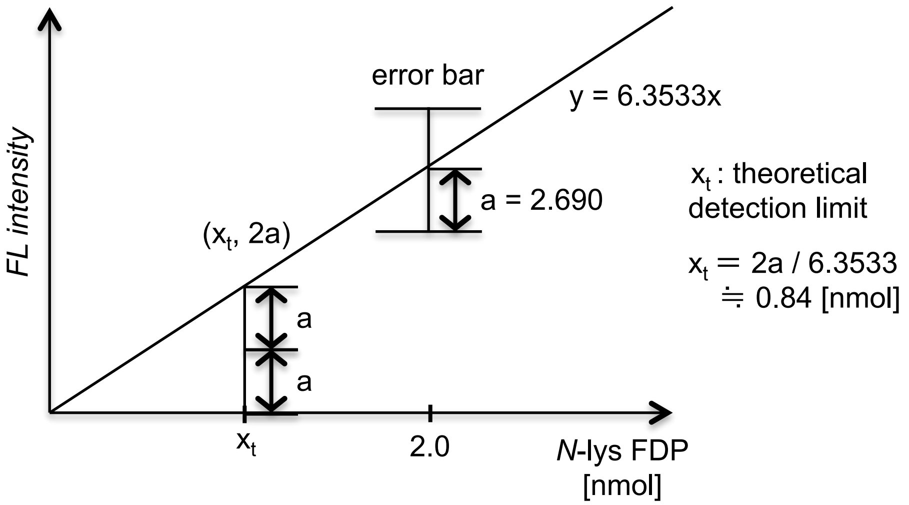

Figure S2. Calculation of FDP detection limit based on standard curve obtained in Figure 4B.

NMR spectrum (^{1}H, ^{13}C)

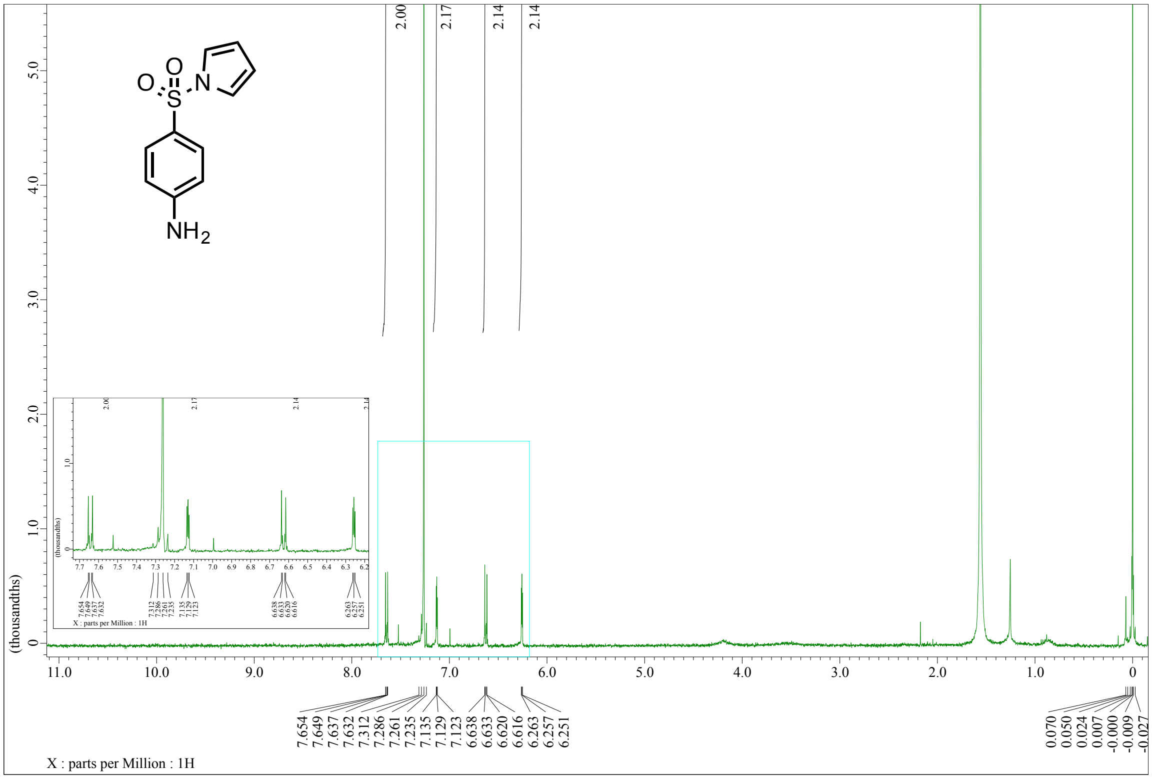

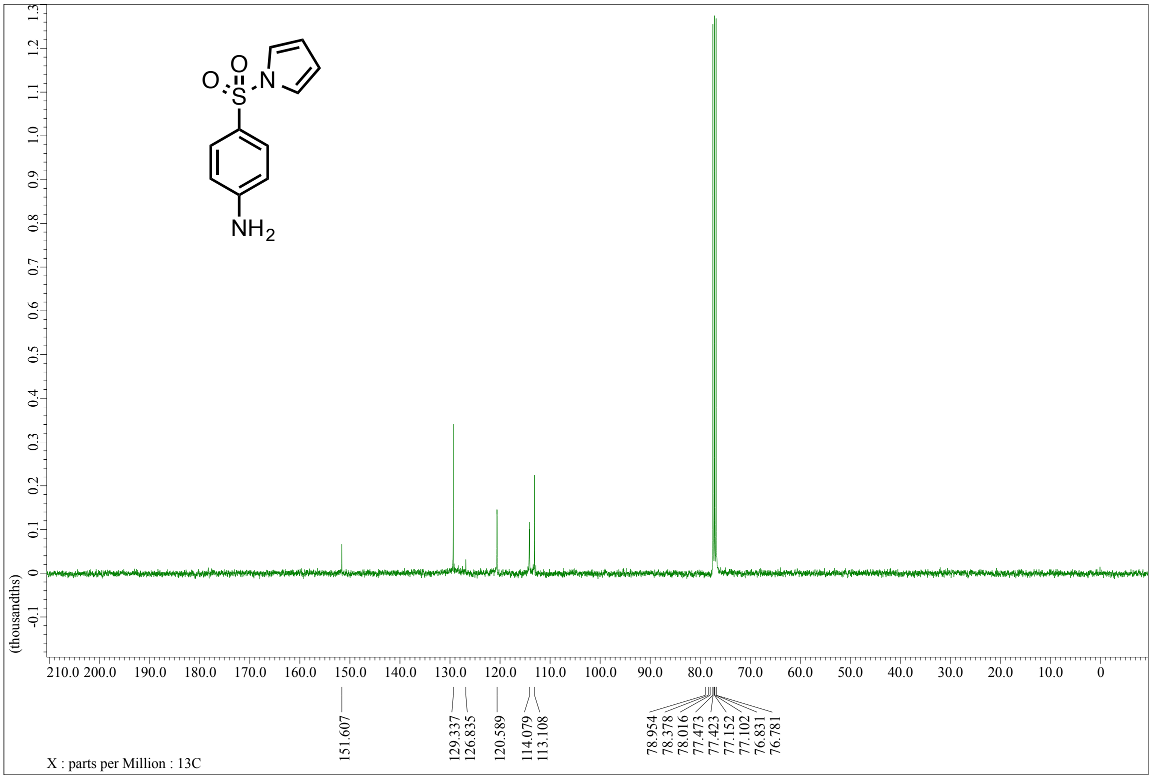

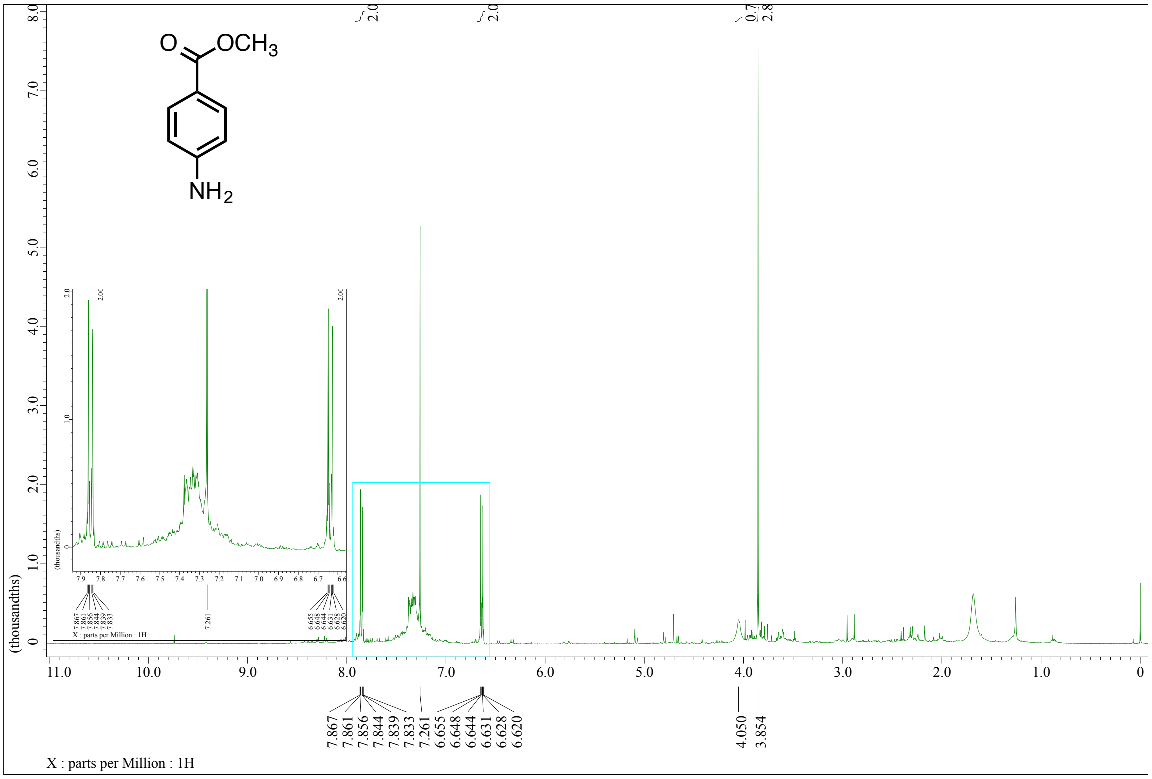
O
OCH3
NH2
(thousandths)
X : parts per Million : 1H
7.867
7.861
7.856
7.844
7.839
7.833
7.261
6.655
6.648
6.644
6.631
6.628
6.620
4.050
3.854
2.0
2.0
0.7
2.8

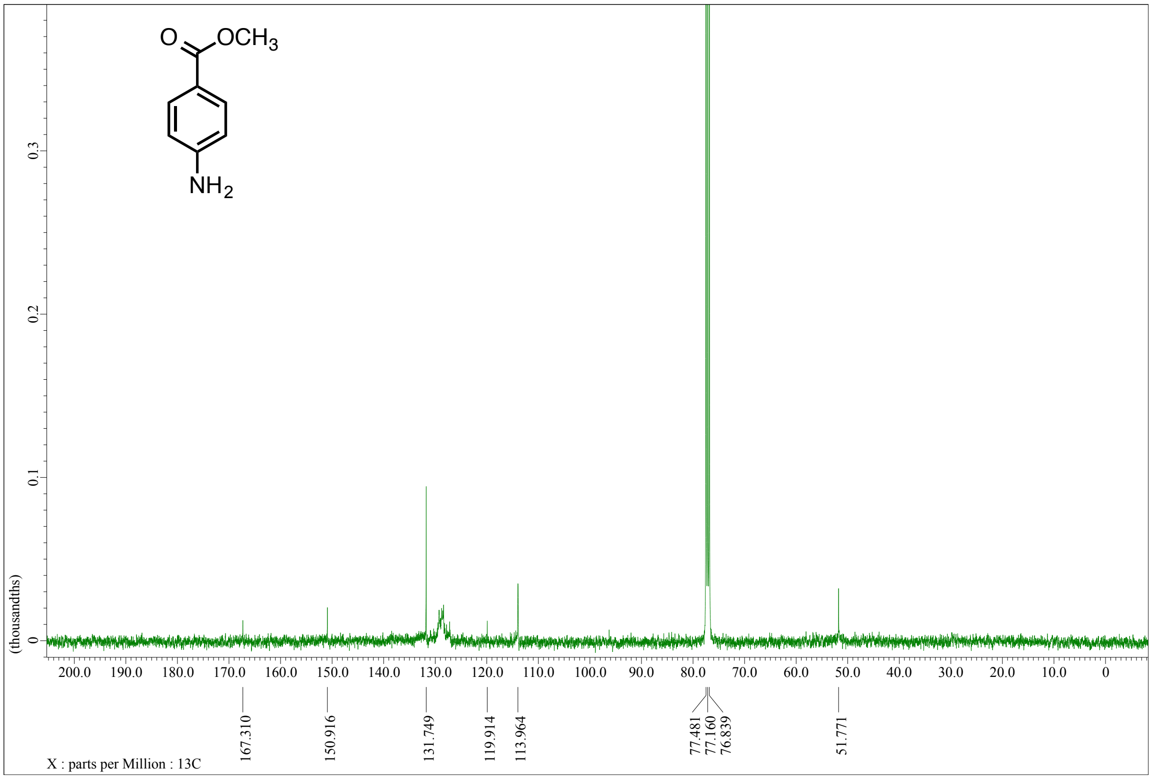
O
OCH3
NH2
(thousandths)
X : parts per Million : 13C
167.310
150.916
131.749
119.914
113.964
77.481
77.160
76.839
51.771

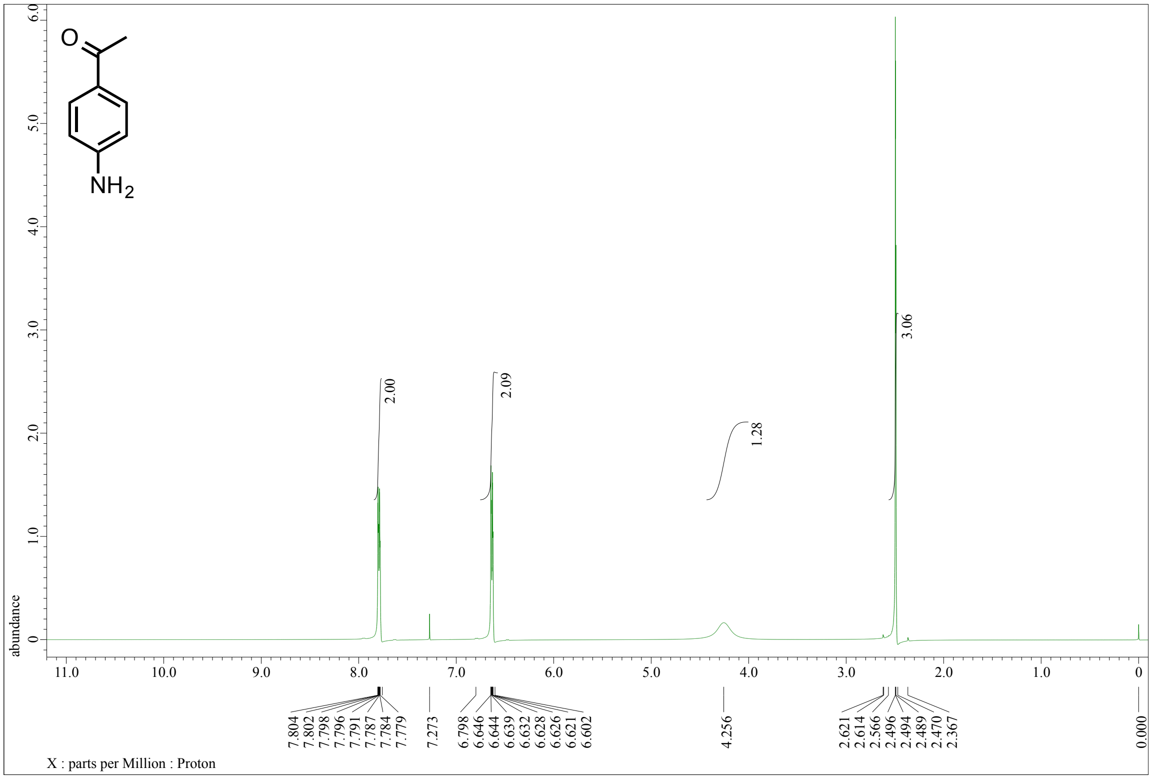

O
NH2
abundance
6.0
5.0
4.0
3.0
2.0
1.0
0
2.00
2.09
1.28
3.06
11.0 10.0 9.0 8.0 7.0 6.0 5.0 4.0 3.0 2.0 1.0 0
7.804
7.802
7.798
7.796
7.791
7.787
7.784
7.779
7.273
6.798
6.646
6.644
6.639
6.632
6.628
6.626
6.621
6.602
4.256
2.621
2.614
2.566
2.496
2.494
2.489
2.470
2.367
0.000
X : parts per Million : Proton

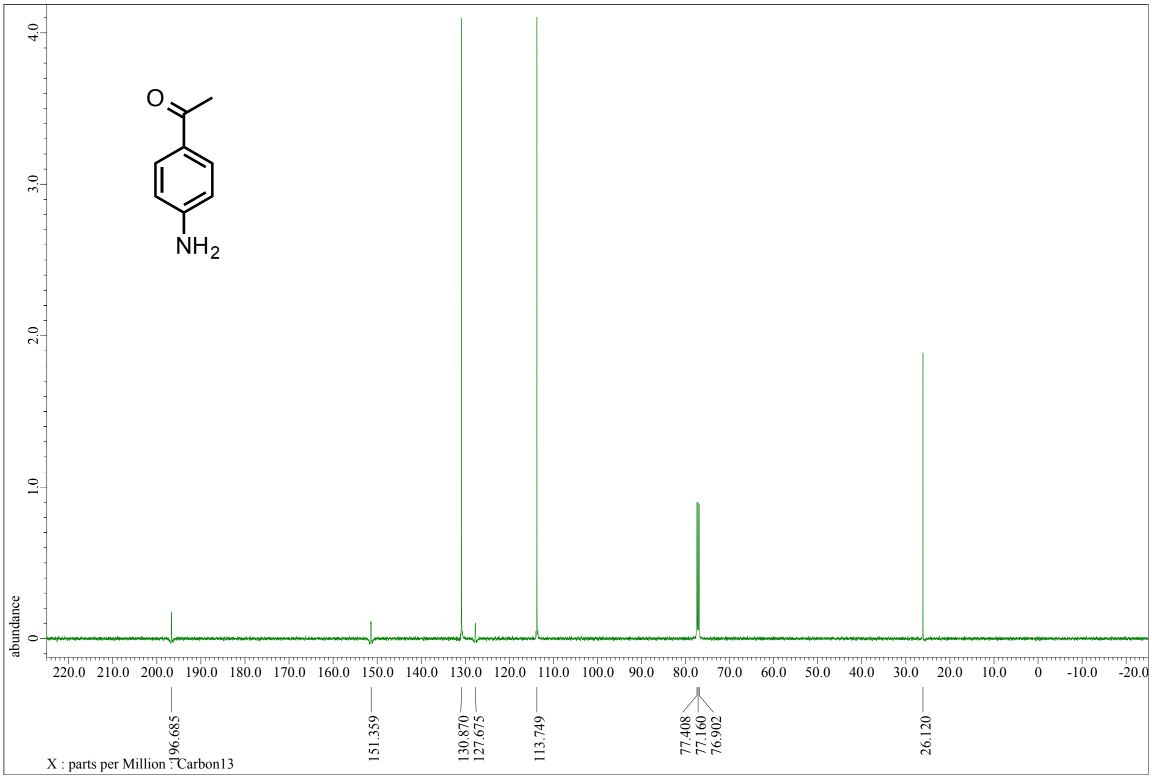

O
NH2
abundance
4.0
3.0
2.0
1.0
0
220.0 210.0 200.0 190.0 180.0 170.0 160.0 150.0 140.0 130.0 120.0 110.0 100.0 90.0 80.0 70.0 60.0 50.0 40.0 30.0 20.0 10.0 0 -10.0 -20.0
196.685
151.359
130.870
127.675
113.749
77.408
77.160
76.902
26.120
X : parts per Million : Carbon13

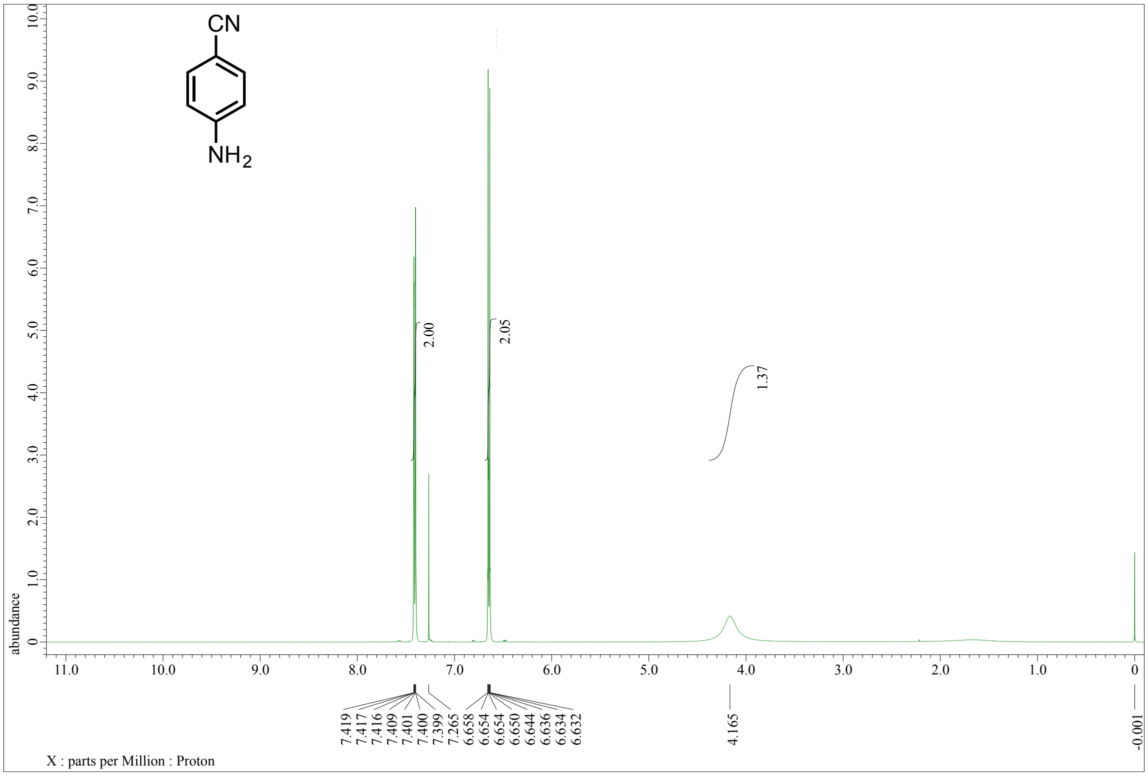
CN
NH2
abundance
X : parts per Million : Proton
2.00
2.05
1.37
7.419
7.417
7.416
7.409
7.401
7.400
7.399
7.265
6.658
6.654
6.654
6.650
6.644
6.636
6.634
6.632
4.165
-0.001

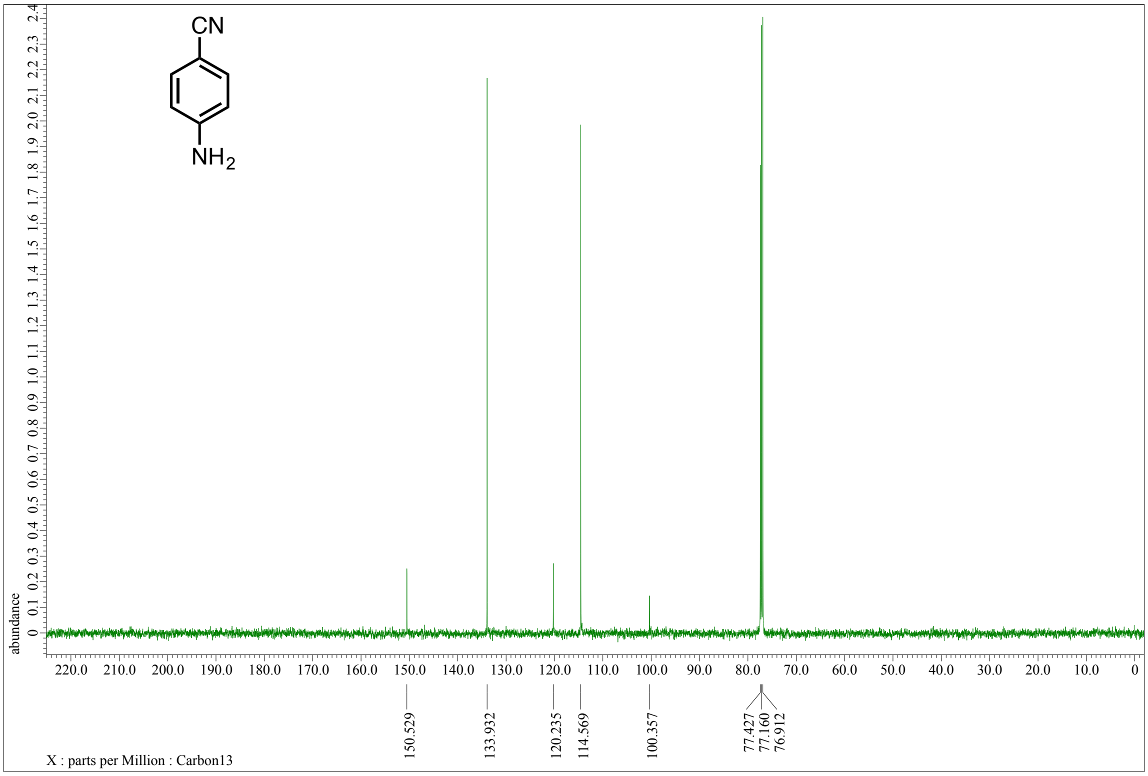
CN
NH2
abundance
X : parts per Million : Carbon13
150.529
133.932
120.235
114.569
100.357
77.427
77.160
76.912

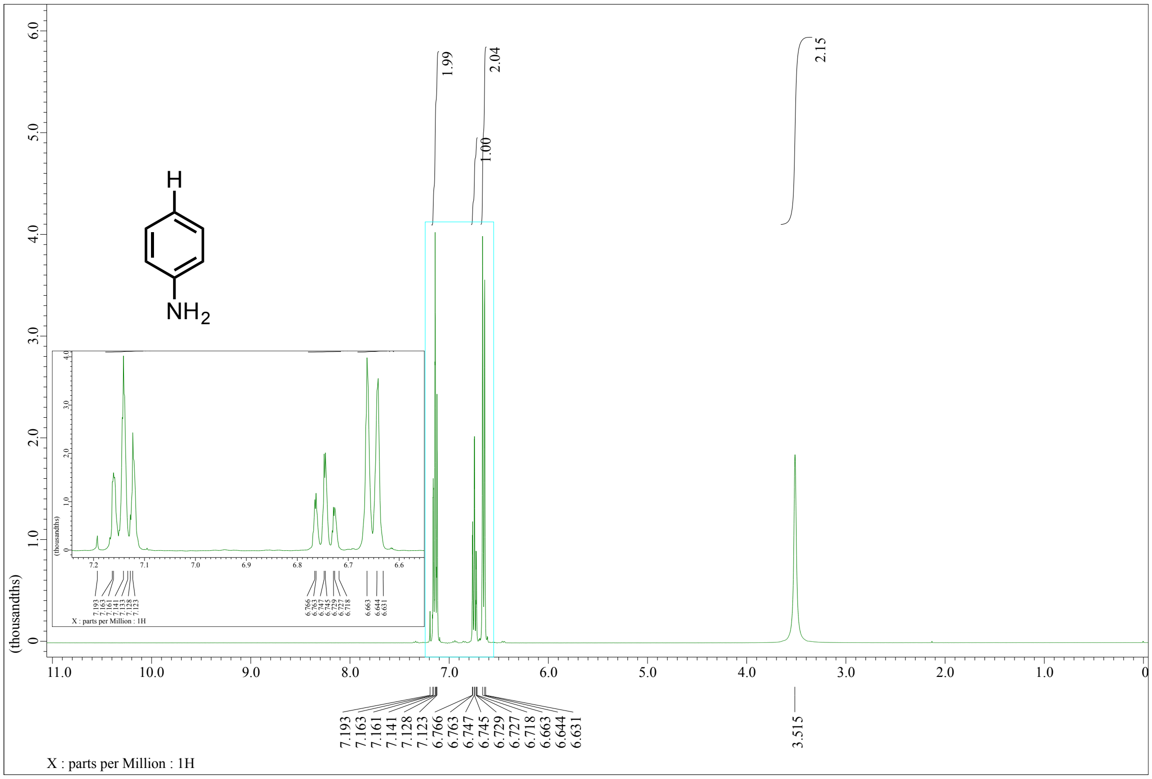
H
NH2
1.99
2.04
1.00
2.15
(thousandths)
X : parts per Million : 1H
7.193
7.163
7.161
7.141
7.128
7.123
6.766
6.763
6.747
6.745
6.729
6.727
6.718
6.663
6.644
6.631
3.515

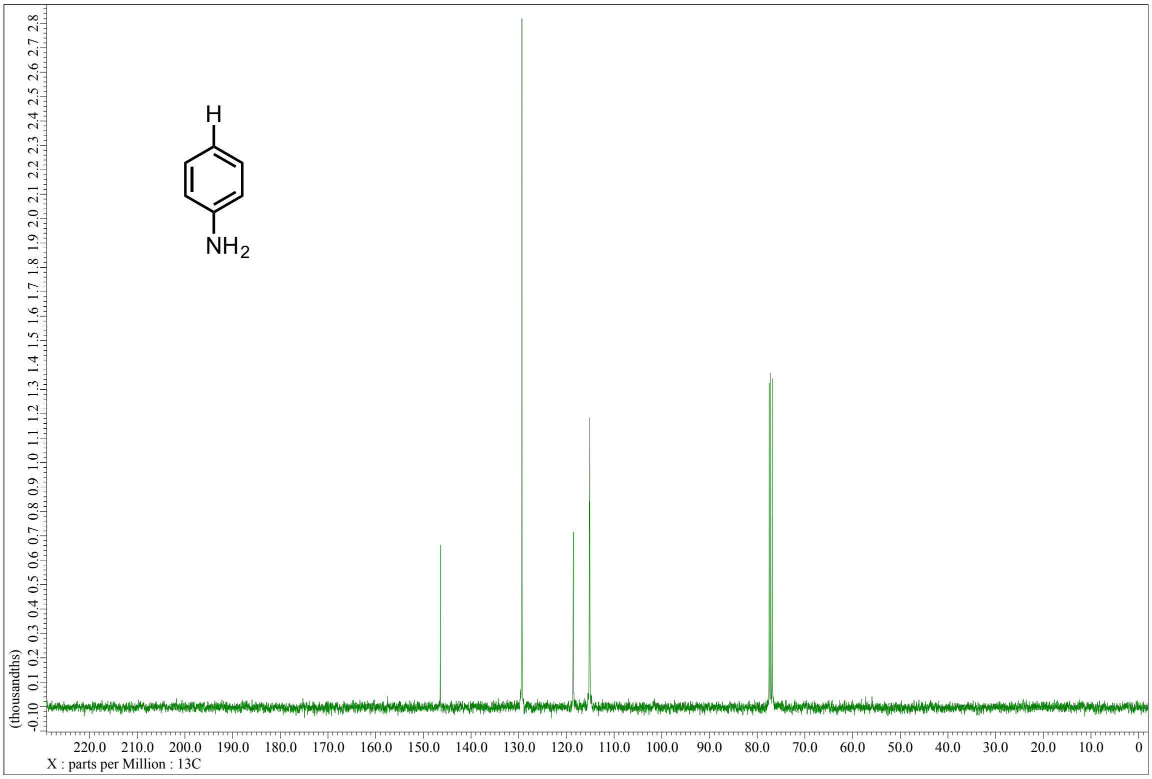
H
NH2
(thousandths)
X : parts per Million : 13C

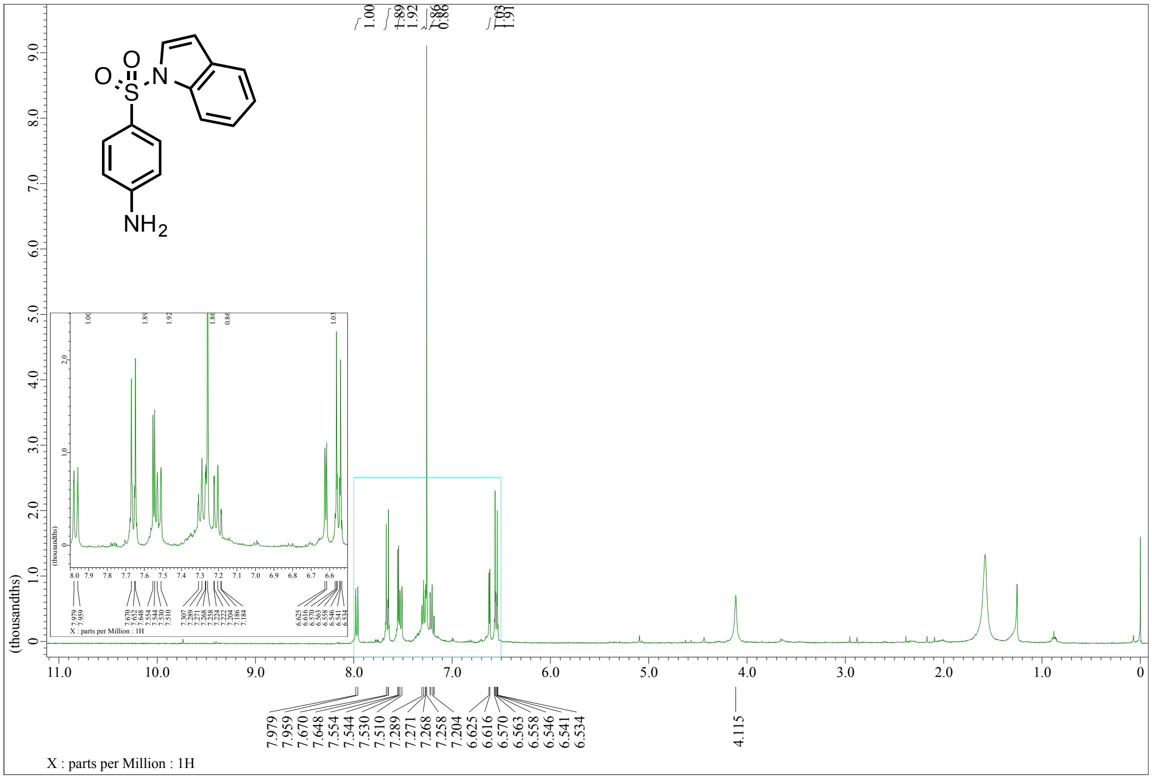
NH2
(thousandths)
X : parts per Million : 1H
1.00
1.89
1.92
1.86
0.86
1.03
1.91
7.979
7.959
7.670
7.648
7.554
7.544
7.530
7.510
7.289
7.271
7.268
7.258
7.204
6.625
6.616
6.570
6.563
6.558
6.546
6.541
6.534
4.115

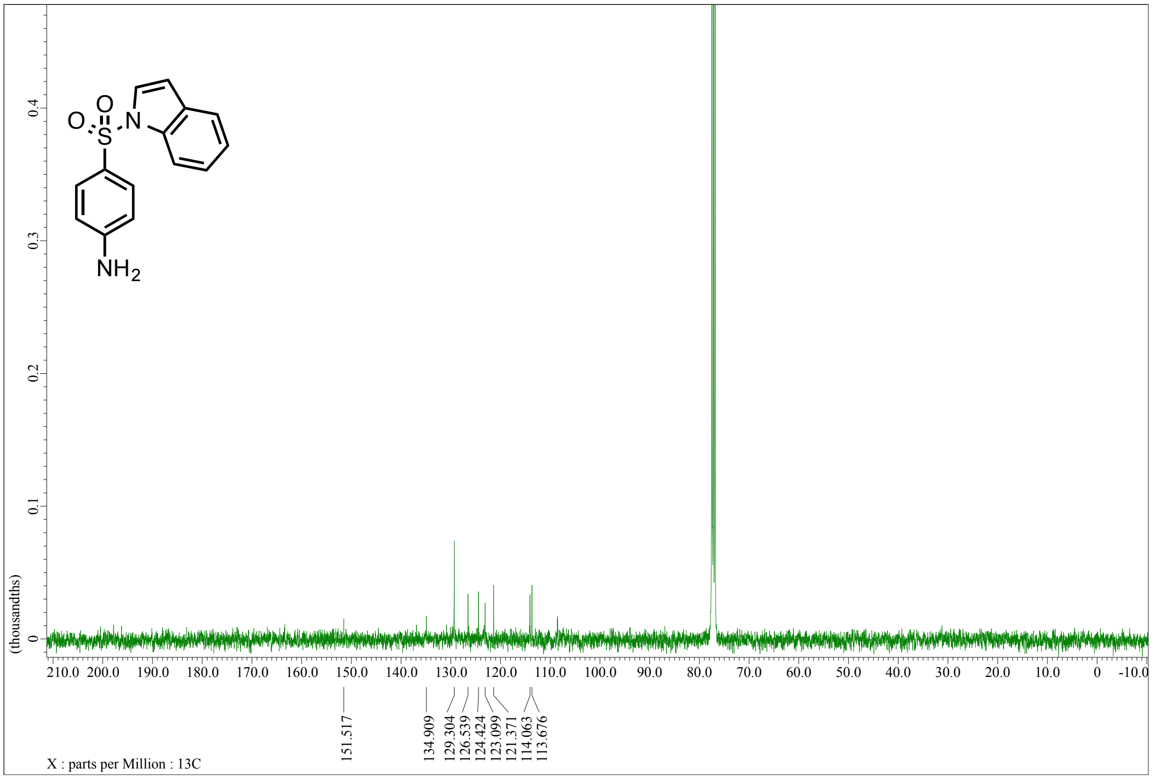
NH2
(thousandths)
X : parts per Million : 13C
151.517
134.909
129.304
126.539
124.424
123.099
121.371
114.063
113.676

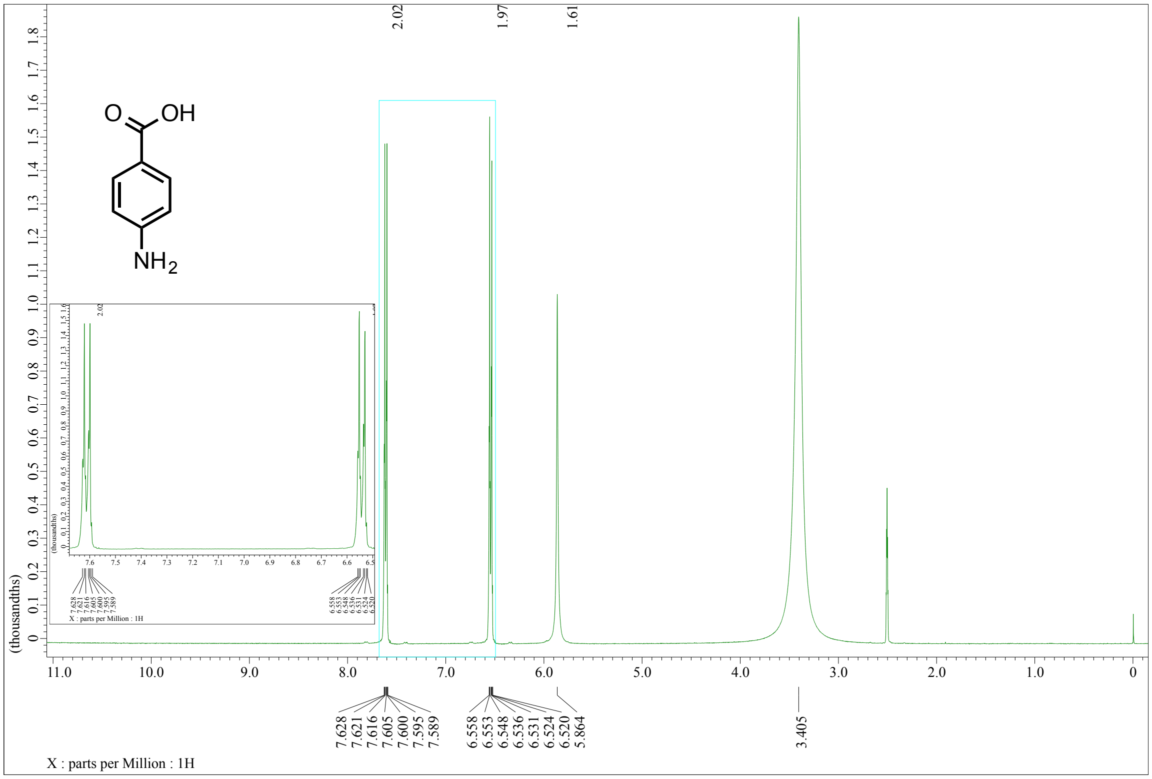

O
OH
NH2
2.02
1.97
1.61
(thousandths)
7.628
7.621
7.616
7.605
7.600
7.595
7.589
6.558
6.553
6.548
6.536
6.531
6.524
6.520
5.864
3.405
X : parts per Million : 1H

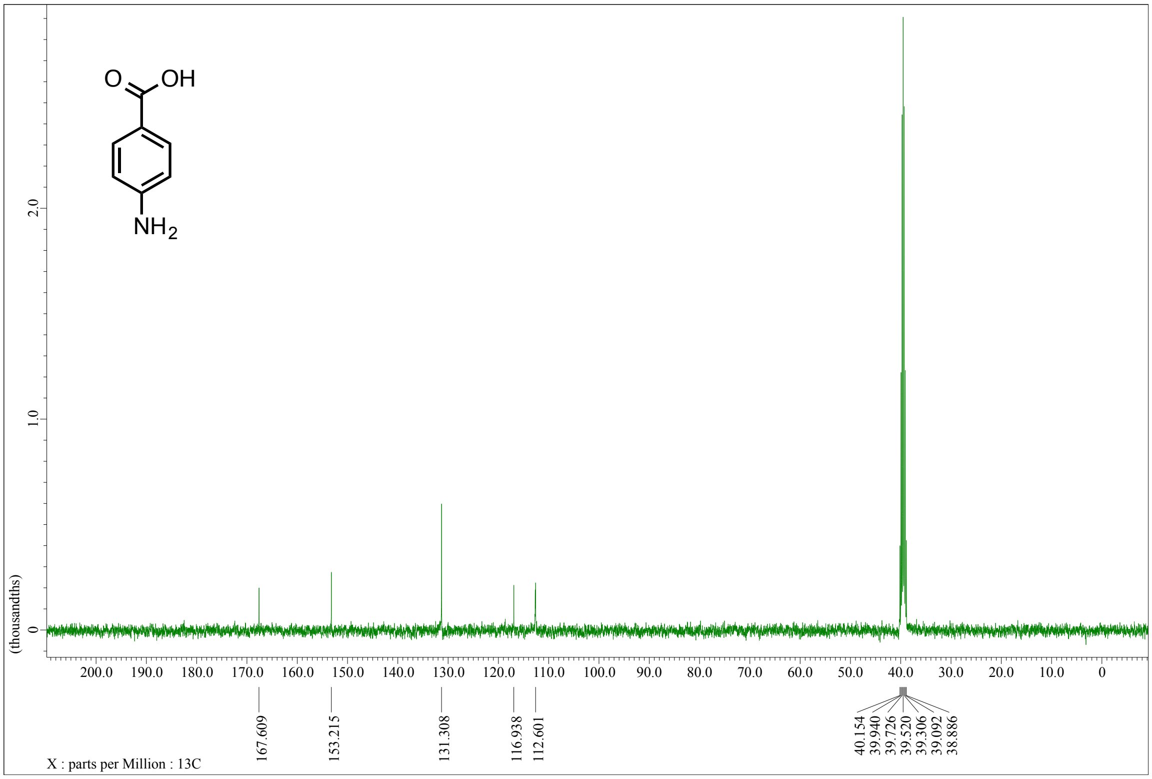

O
OH
NH2
(thousandths)
167.609
153.215
131.308
116.938
112.601
40.154
39.940
39.726
39.520
39.306
39.092
38.886
X : parts per Million : 13C

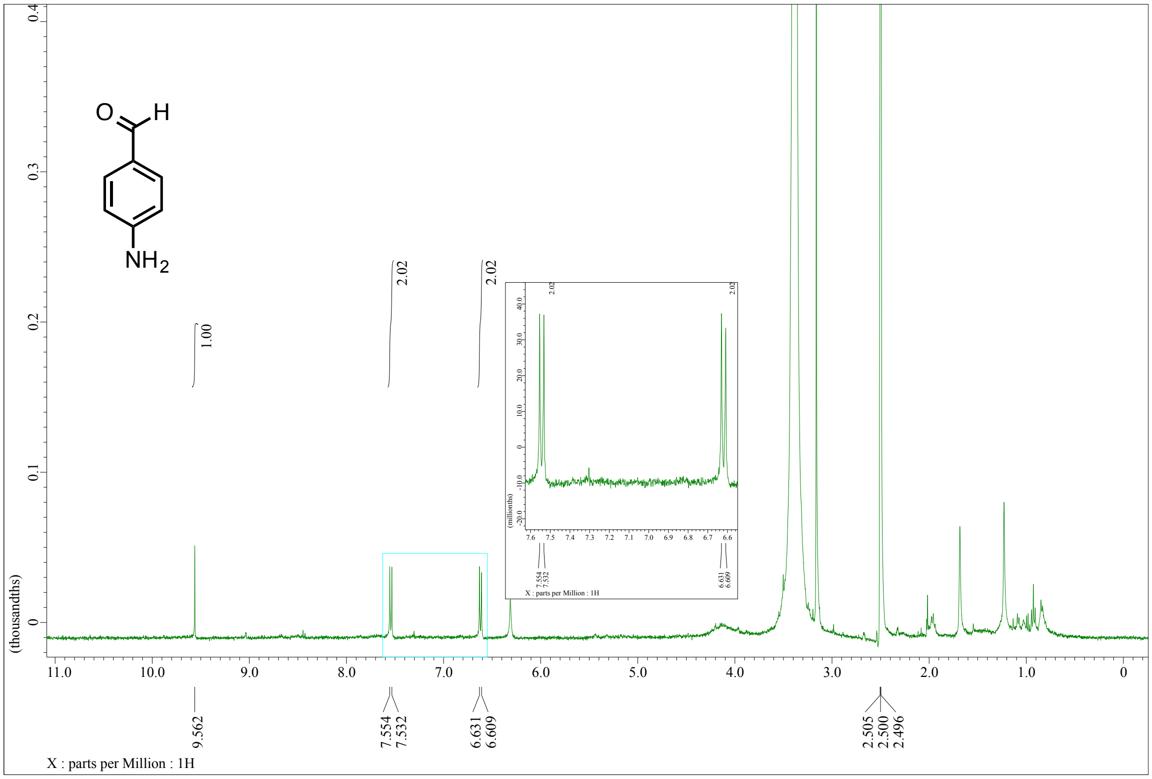
O
H
NH2
1.00
2.02
2.02
(thousandths)
9.562
7.554
7.532
6.631
6.609
2.505
2.500
2.496
X : parts per Million : 1H
(millionths)
X : parts per Million : 1H

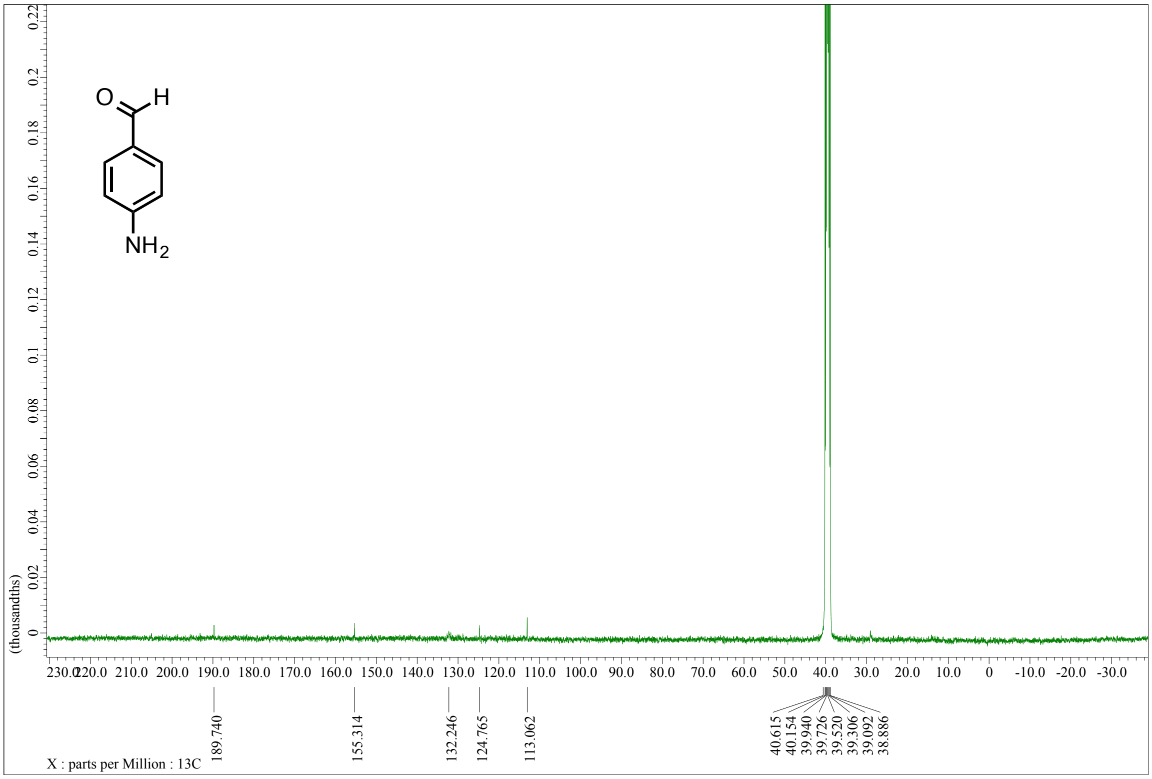
O
H
NH2
(thousandths)
189.740
155.314
132.246
124.765
113.062
40.615
40.154
39.940
39.726
39.520
39.306
39.092
38.886
X : parts per Million : 13C

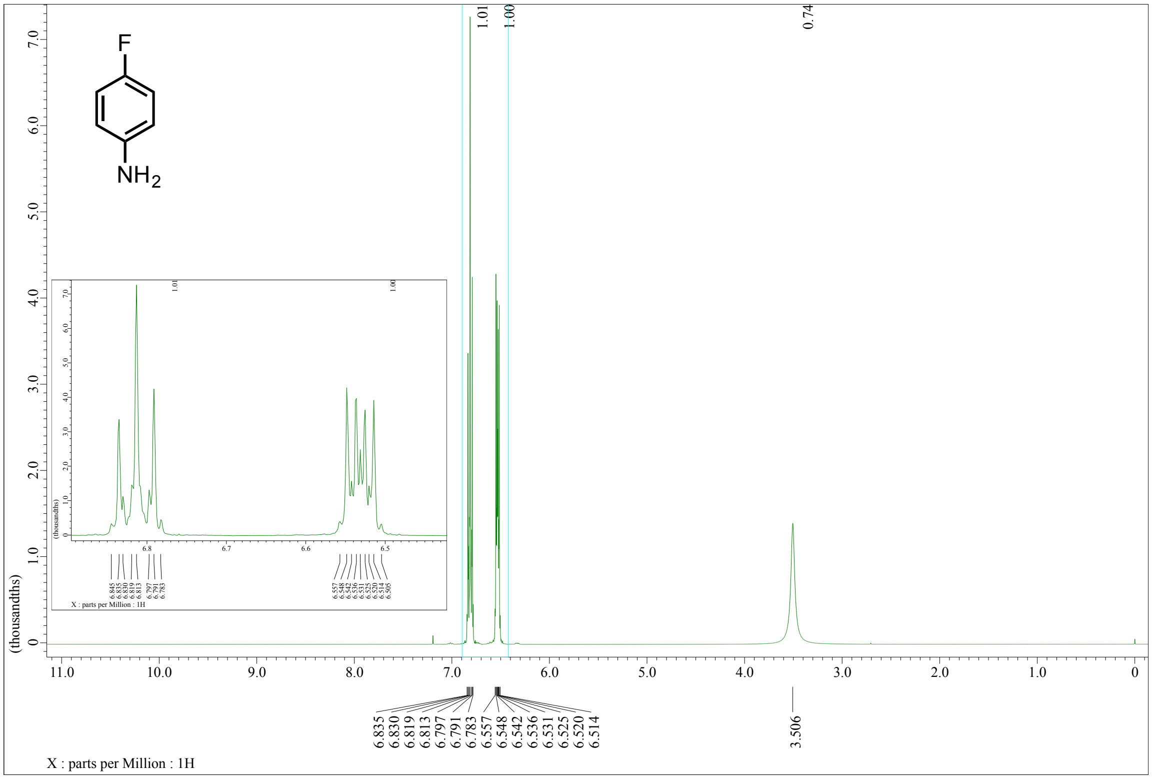
F
NH2
1.01
1.00
0.74
(thousandths)
6.835
6.830
6.819
6.813
6.797
6.791
6.783
6.557
6.548
6.542
6.536
6.531
6.525
6.520
6.514
3.506
X : parts per Million : 1H

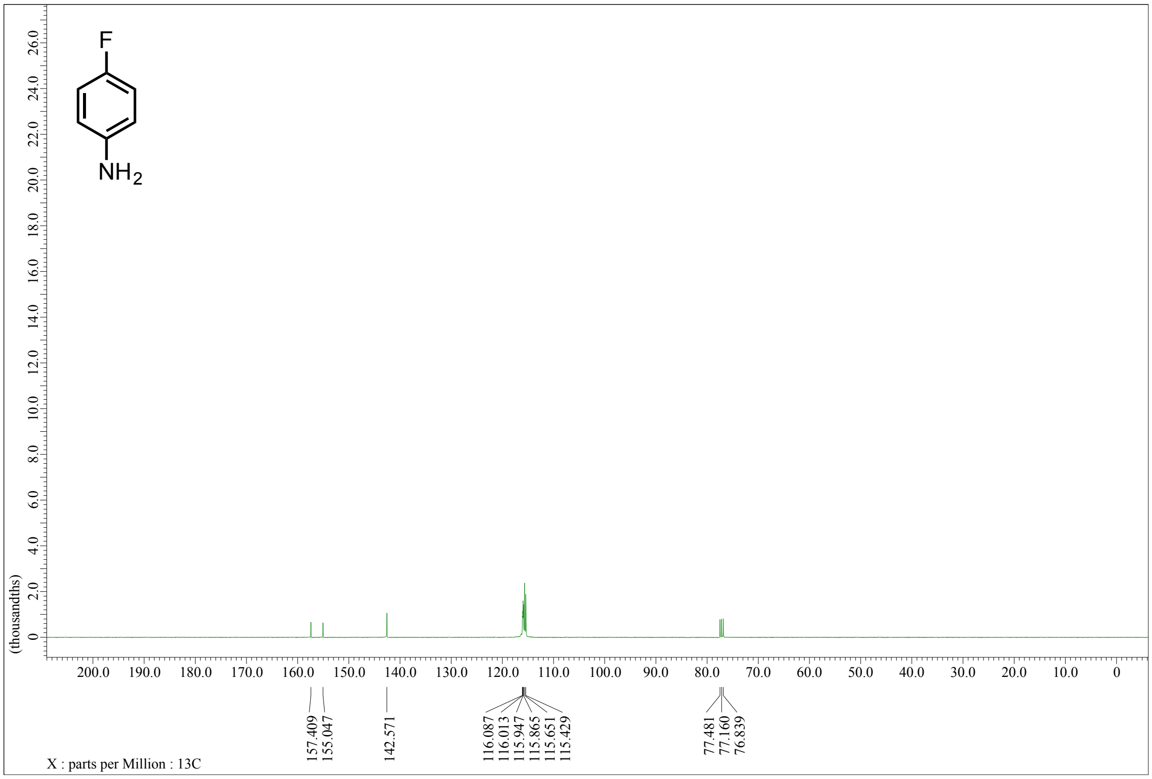
F
NH2
(thousandths)
157.409
155.047
142.571
116.087
116.013
115.947
115.865
115.651
115.429
77.481
77.160
76.839
X : parts per Million : 13C

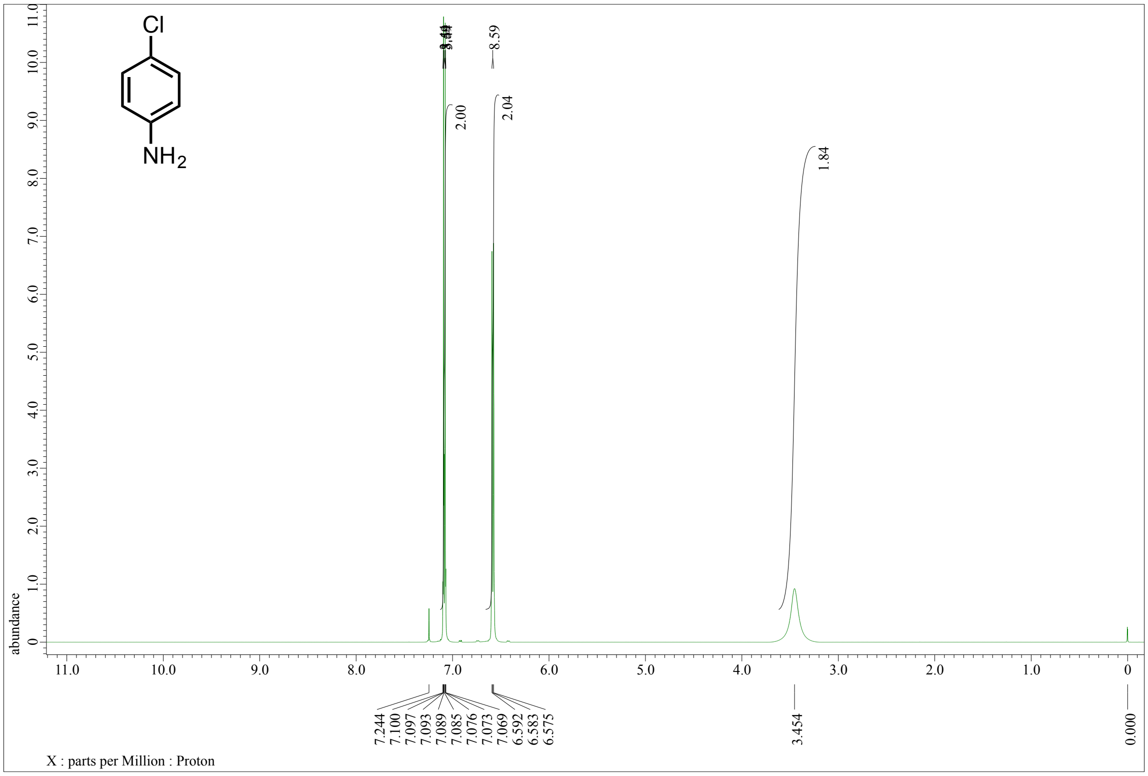

Cl
NH2
abundance
X : parts per Million : Proton
7.244
7.100
7.097
7.093
7.089
7.085
7.076
7.073
7.069
6.592
6.583
6.575
3.454
0.000
2.00
2.04
1.84
8.59

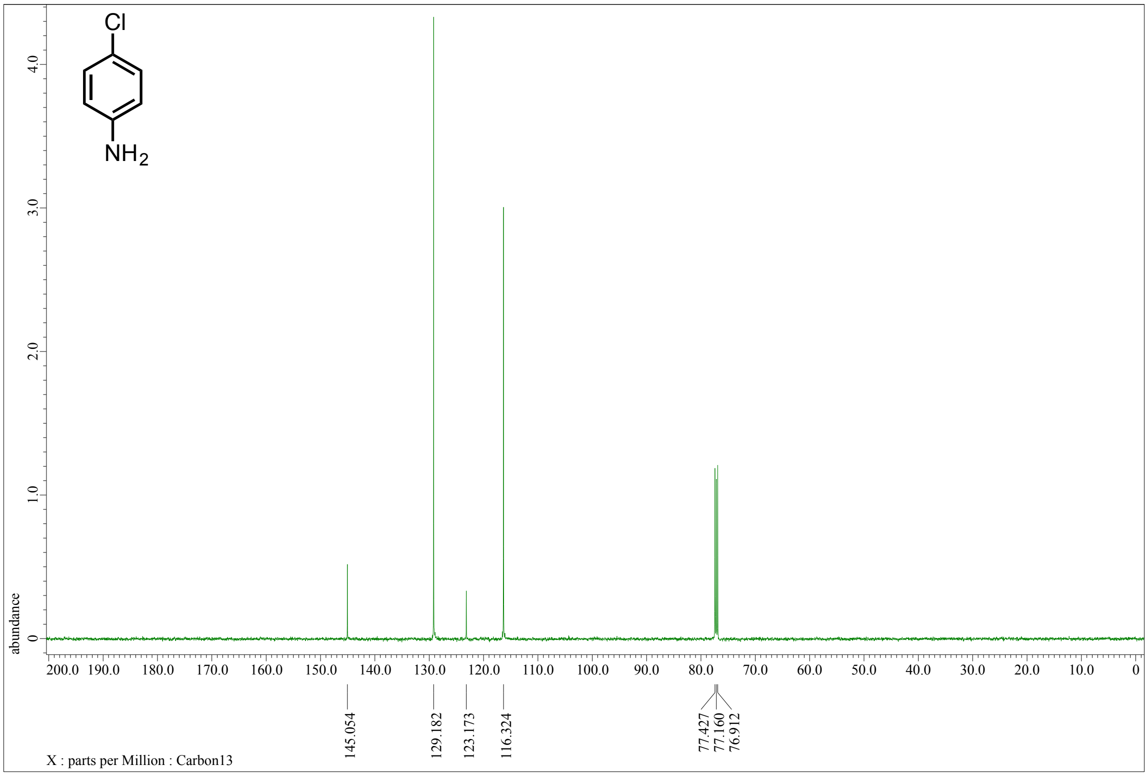

Cl
NH2
abundance
X : parts per Million : Carbon13
145.054
129.182
123.173
116.324
77.427
77.160
76.912

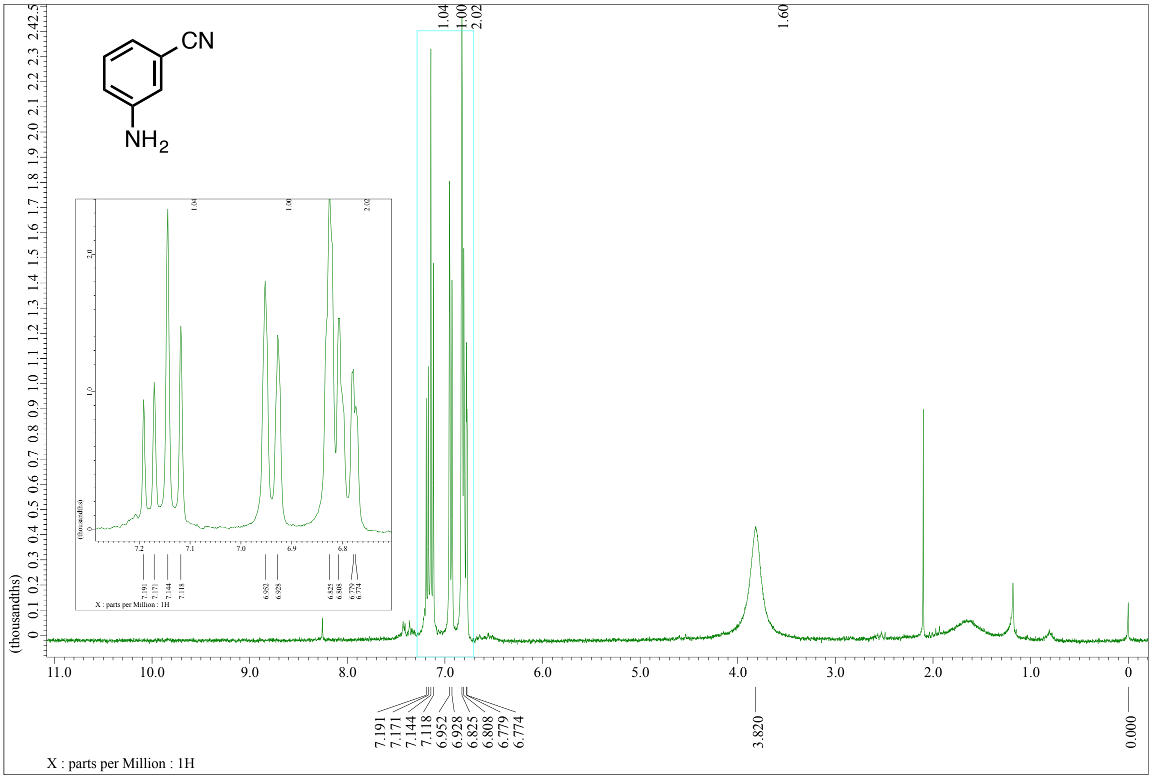
CN
NH2
1.04
1.00
2.02
1.60
(thousandths)
7.191
7.171
7.144
7.118
6.952
6.928
6.825
6.808
6.779
6.774
3.820
0.000
X : parts per Million : 1H

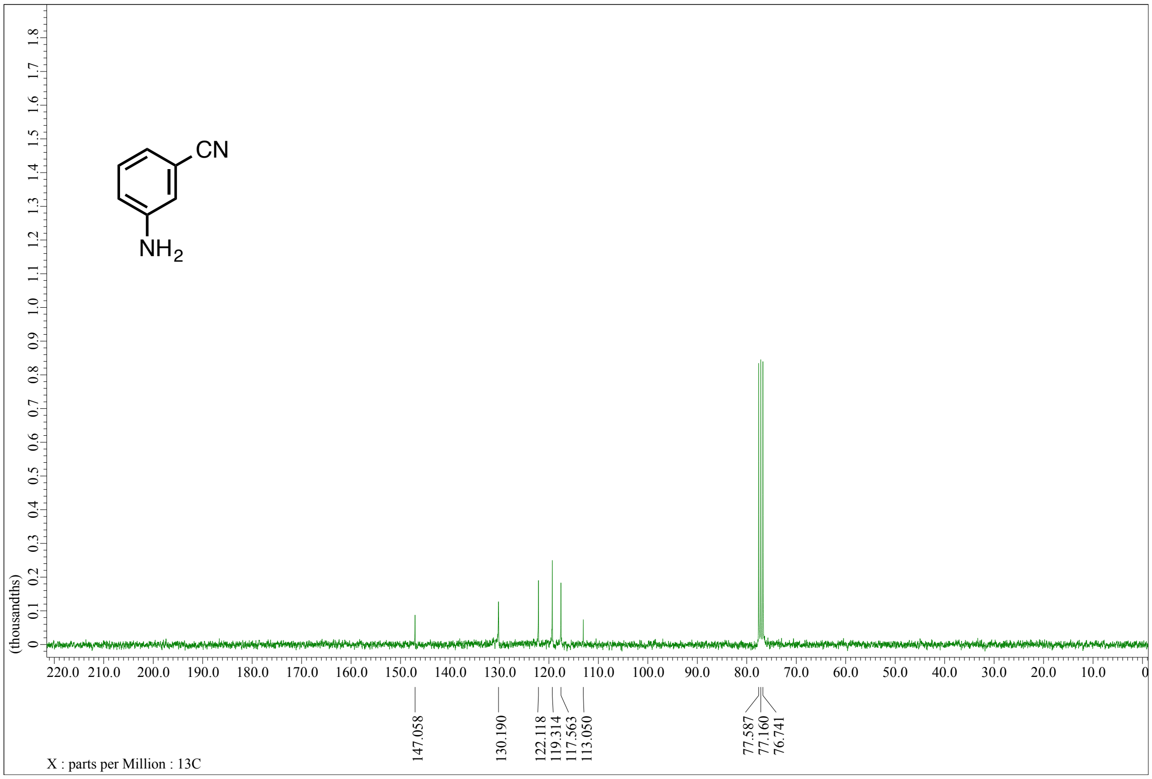
CN
NH2
(thousandths)
147.058
130.190
122.118
119.314
117.563
113.050
77.587
77.160
76.741
X : parts per Million : 13C

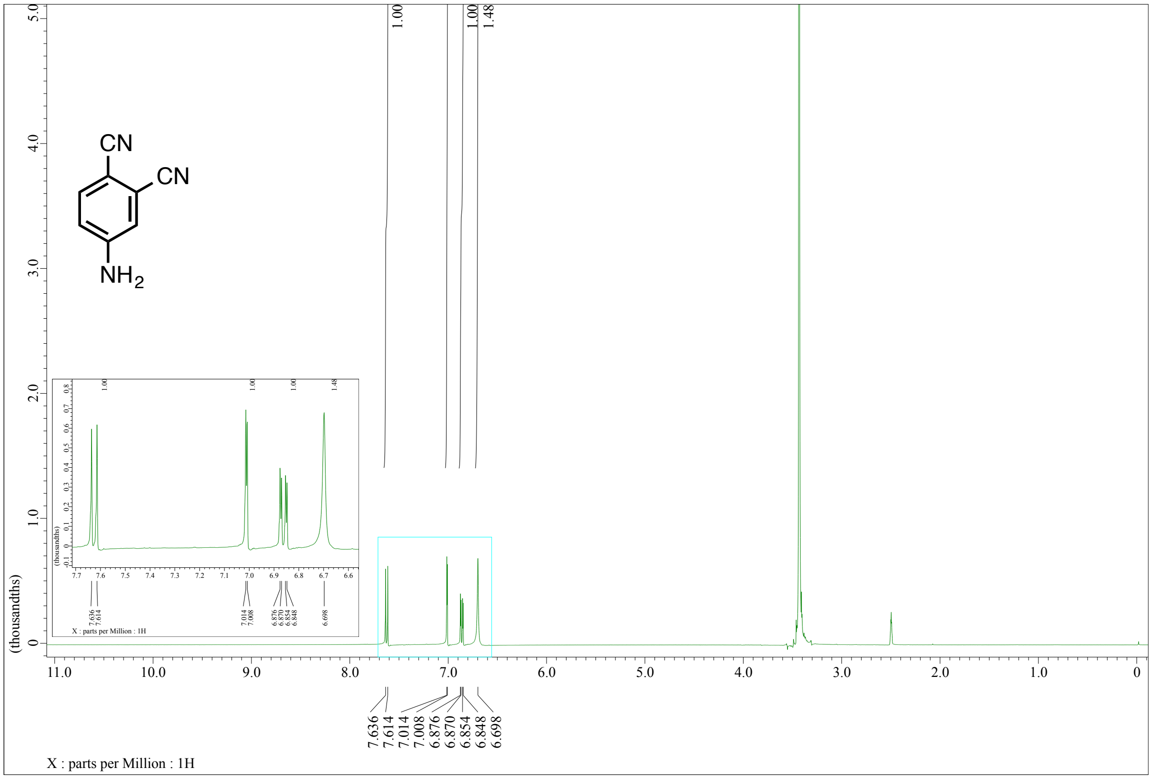
CN
CN
NH2
(thousandths)
X : parts per Million : 1H
7.636
7.614
7.014
7.008
6.876
6.870
6.854
6.848
6.698
1.00
1.00
1.48

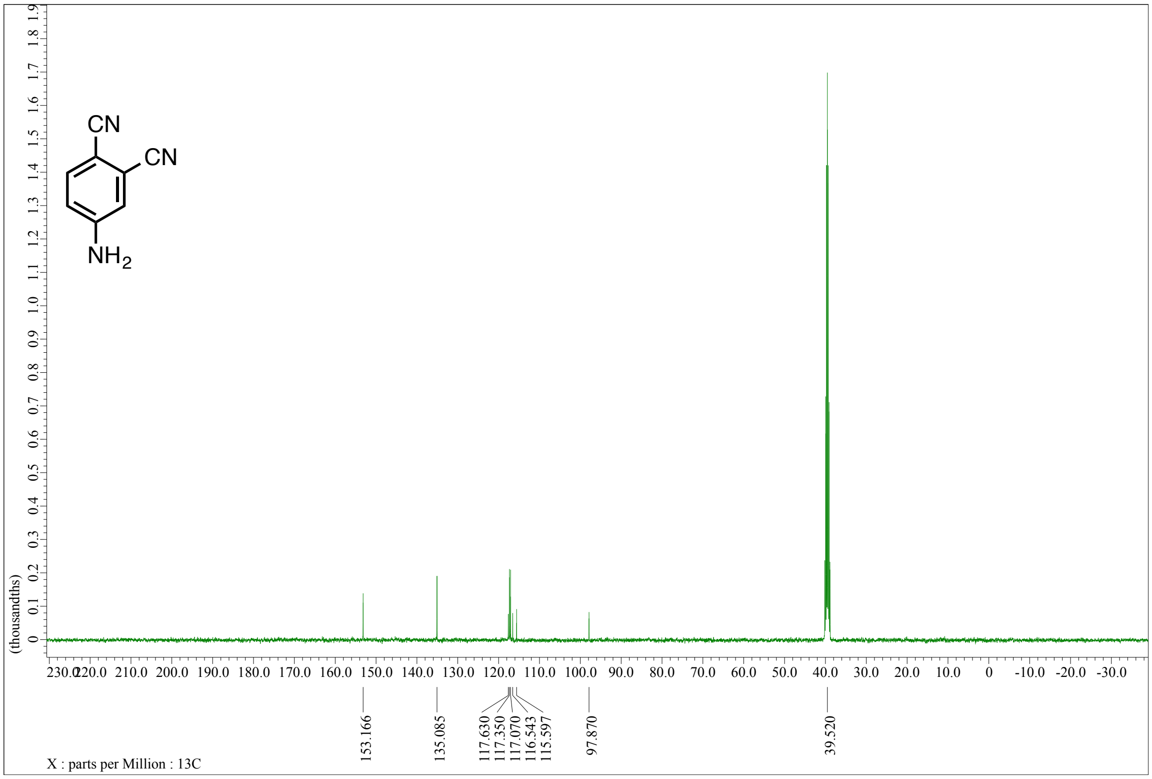
CN
CN
NH2
(thousandths)
X : parts per Million : 13C
153.166
135.085
117.630
117.350
117.070
116.543
115.597
97.870
39.520
